# Exposure to halogenated ethers causes neurodegeneration and behavioural changes in young healthy experimental animals: a systematic review and meta analyses

**DOI:** 10.1038/s41598-023-35052-4

**Published:** 2023-05-18

**Authors:** Carlijn R. Hooijmans, Marije Buijs, Frederique Struijs, Thijs Som, Najma Karim, Gert-Jan Scheffer, Ignacio Malagon

**Affiliations:** Department of Anesthesiology, Pain and Palliative Medicine, Radboud University Medical Center, Geert Grooteplein-Noord 21, route 126, 6525 GA Nijmegen, The Netherlands

**Keywords:** Experimental models of disease, Preclinical research, Translational research

## Abstract

The FDA issued a warning that repeated and prolonged use of inhalational anaesthetics in children younger than 3 years may increase the risk of neurological damage. Robust clinical evidence supporting this warning is however lacking. A systematic review of all preclinical evidence concerning isoflurane, sevoflurane, desflurane and enflurane exposure in young experimental animals on neurodegeneration and behaviour may elucidate how severe this risk actually is PubMed and Embase were comprehensively searched on November 23, 2022. Based on predefined selection criteria the obtained references were screened by two independent reviewers. Data regarding study design and outcome data (Caspase-3 and TUNEL for neurodegeneration, Morris water maze (MWM), Elevated plus maze (EPM), Open field (OF) and Fear conditioning (FC)) were extracted, and individual effect sizes were calculated and subsequently pooled using the random effects model. Subgroup analyses were predefined and conducted for species, sex, age at anesthesia, repeated or single exposure and on time of outcome measurement. Out of the 19.796 references screened 324 could be included in the review. For enflurane there were too few studies to conduct meta-analysis (n = 1). Exposure to sevoflurane, isoflurane and desflurane significantly increases Caspase-3 levels and TUNEL levels. Further, sevoflurane and isoflurane also cause learning and memory impairment, and increase anxiety. Desflurane showed little effect on learning and memory, and no effect on anxiety. Long term effects of sevoflurane and isoflurane on neurodegeneration could not be analysed due to too few studies. For behavioural outcomes, however, this was possible and revealed that sevoflurane caused impaired learning and memory in all three related outcomes and increased anxiety in the elevated plus maze. For isoflurane, impaired learning and memory was observed as well, but only sufficient data was available for two of the learning and memory related outcomes. Further, single exposure to either sevoflurane or isoflurane increased neurodegeneration and impaired learning and memory. In summary, we show evidence that exposure to halogenated ethers causes neurodegeneration and behavioural changes. These effects are most pronounced for sevoflurane and isoflurane and already present after single exposure. To date there are not sufficient studies to estimate the presence of long term neurodegenerative effects. Nevertheless, we provide evidence in this review of behavioral changes later in life, suggesting some permanent neurodegenerative changes. Altogether, In contrast to the warning issued by the FDA we show that already single exposure to isoflurane and sevoflurane negatively affects brain development. Based on the results of this review use of sevoflurane and isoflurane should be restrained as much as possible in this young vulnerable group, until more research on the long term permanent effects have been conducted.

## Introduction

In 2016 the U.S. Food Drug Administration (FDA) warned that lengthy (more than 3 h) or repeated use of anesthetics and sedation drugs may negatively affect brain development in children less than 3 years of age (drug safety communication www.fda.gov/drugs/drugsafety/ucm532356.htm). The list of potentially harmful general anesthetics generated by the FDA contains drugs that block *N*-methyl-D-aspartate (NMDA) receptors and/or potentiate gamma-aminobutyric acid (GABA) activity such as the inhalational anesthetics such as isoflurane, sevoflurane, desflurane, etomidate, halothane, ketamine, lorazepam, methohexital, midazolam, pentobarbital and propofol. The evidence underlying this warning contained both clinical and non-clinical data and was very heterogenous in both the design of the studies and the results.

Roughly 50% of the epidemiological literature reviewed showed an association between pediatric exposure and neurodevelopmental outcomes, in particular cognitive and behavioral problems, including neurodevelopmental delay-related diagnoses, learning disabilities, and attention deficit hyperactivity disorder^1–5^. However, it was impossible to determine causality as the reviewed observational studies suffered from many limitations such as (1) heterogeneous exposure and definitions of outcome measures (2) incomplete control of confounding and (3) insufficient power.

More recent evidence from clinical trials is inconclusive as well. The General Anesthesia Compared to Spinal Anesthesia (GAS) trial and results from the Pediatric Anesthesia NeuroDevelopment Assessment (PANDA) Study showed no difference in neuropsychologic scores or neurodevelopmental scores^6–8^, whereas the population based study by Schneuer in 2019 showed that children exposed to general anesthesia before 4 years have poorer development at school entry and school performance^9^. Besides being inconclusive the results of these trials are also limited in their generalizability. None of these trials studied the impact of long duration of general anesthesia exposure. In addition, the PANDA and GAS trial were specific to a single indication and type of surgery.

Regarding, the evidence from preclinical studies, the FDA concluded that the clinical significance of the nonclinical (animal) findings was not known (drug safety communication www.fda.gov/drugs/drugsafety/ucm532356.htm) They, however, did not assess all available preclinical animal evidence. The FDA referred to nineteen animal studies of which 2 were actually reviews^10,11^, ten studies investigated the effects of inhalational anesthetics such as ketamine, midazolam or propofol^12–21^, and only seven studies investigated the effects of various halogenated ethers^22–28^.

Liu et al. conducted a systematic review on inhalational anesthetics in 2013^29^. But the published search strategy seems to miss some important keywords (such as the individual anesthetic agent terms) increasing the risk of missing potential relevant studies in the analysis. In addition, not all formal steps of a systematic review are conducted (e.g. formal screening based on eligibility criteria, risk of bias assessment etc.). Finally, Liu et al. did not present a quantitative summary of the results (e.g. meta analysis) which would be very useful to obtain a clear overview of the potential risks of inhalation anesthetics.

Altogether, robust clinical evidence supporting the warning issued by the FDA is lacking and the analyses conducted so far of the experimental animal data appears to be incomplete. In order to unravel whether or not there is robust preclinical evidence to substantiate the warning by the FDA that halogenated ethers may negatively affect brain development, we present a formal systematic review and meta analysis of the effects of halogenated ethers (sevoflurane, isoflurane, enflurane and desflurane) on neurodegeneration and behavioral changes in the developing animal brain.

## Methods

This systematic review investigated the effects inhalational anaesthetics (isoflurane, desflurane, sevoflurane and enflurane) on neurodegeneration and behavioural changes in young experimental animals. The review methodology was specified in advance and published in PROSPERO [CRD42020220146] and conducted according to guidelines for preclinical meta analysis^30,31^. This systematic review is reported according to the Preferred Reporting Items for Systematic Reviews and Meta-Analyses (PRISMA 2020) statement^32^.

### Paper identification and selection

We performed a systematic, search in Medline through the PubMed interface and EMBASE to identify all the studies regarding the effects inhalational anaesthetics on neurodegeneration and behavioural changes in young experimental animals. The full search strategy (Supplemental file 1) was based on the search components ‘inhalation anaesthetic, infant, animal’^33–35^. Search results from both databases were combined and duplicates were removed using Endnote software initially and thereafter manually. The initial search was performed on 18th of March 2020 and updates were done at the 10th of November 2020, and 23rd of November 2022.

Studies were excluded when they met at least one of the following criteria: (1) the study was not an original study, (2) the study did not use an in vivo animal model, (3) Other inhalational anesthetics than isoflurane, desflurane, sevoflurane or enflurane were administered, (4) the study included animals that reached sexual maturity.

During full text screening studies were excluded when they met at least one of the previous criteria or one of the following criteria: (5) the study did not assess the effects of inhalational anesthetics on neurodegeneration, cognitive impairment or other behavioral changes, (6) the animals used in the study underwent a cointervention, (7) no or incorrect control groups are used or (8) the animals used in the study suffered from comorbidities.

As the warning for the FDA was focussed on 0–3 year old children we defined a corresponding age per species. As it was very challenging to literally translate per species to an age between 0 and 3 years we decided to focuses on a age which represents “children”/ right before sexual maturity. In mice and rats the age limit was set on 60 days^36–38^ in rhesus macaque monkeys 5 years^39^, and in rabbits 28 was days^40^ was set as the maximum age limit.

Neurodegeneration was defined as a test directly measuring cell death.

Eros screening software was used for the references retrieved in the initial search, and Rayyan screening software for the references retrieved during the update search since the EROS software was during the update no longer accessible, as a consequence reasons for exclusions could not have been made transparent. References were screened by two independent reviewers (either MB, TS, NK). Disagreements were resolved by consensus after discussion with the third researcher.

### Study characteristics and outcome data extraction

For all included studies, the following characteristics were extracted: publication (author + year), species, strain, age at intervention, administration route, dose, dose frequency, duration intervention, outcome measures regarding neurodegeneration and outcome measures regarding behavioural tests.

The data from the following outcomes were extracted for meta analyses: caspase-3, TUNEL, Morris water maze, cued fear conditioning, contextual fear conditioning, open field test and elevated plus maze. Supplemental file 2 provides insight in the extracted parameters per behavioural test. The mean, SD and the n were extracted for all independent comparisons. Data represented in SEM or median were also included and were recalculated using SD = SE*√n and medians were recalculated using the formulas from Hozo et al. 2005^41^. In case it was not reported if the variance was reported in SD or SE, it was assumed to be SE in order to keep the results conservative. When the number of animals (n) was given as a range (e.g. n = 6–8) the lowest number was selected.

In case of repeated measurements, the measurements with the largest effect was taken.

When data were only presented graphically, they were measured using FIJI an image processing program, by two independent reviewers. When outcome measure data were missing, we attempted to contact authors for additional information (a maximum of two emails were sent). When the data could not be obtained, a conservative estimate was used if possible.

### Data-analysis

The extracted data was analyzed using the software Comprehensive Meta-Analysis (CMA) Version 3.0. First, we calculated the effect size (Hedges g) for each individual comparison.

When multiple experimental groups were compared to the same control group, the group size of the control group was corrected for the number of comparisons made (n/number of comparisons).

Subsequently we conducted meta-analyses. Despite anticipated heterogeneity, the individual effect sizes were pooled to obtain an overall hedges G and a 95% confidence interval (CI). We used the random effects model^42^, which takes into account the precision of individual studies and the variation between studies and weights each study accordingly.

In case of repeated measures in the intervention group (for example measurements after 30 and 60 min), the largest effect size was selected for each comparison.

I^2^ was used to determine the level of between study heterogeneity.

Subgroups were predefined and registered in a protocol (see PROSPERO [CRD42020220146]). Subgroup analyses were planned for species, sex, age at anesthesia, repeated or single exposure and on timing of outcome measurement relative to the exposure.

Subgroups for the age of the animals was divided into three groups based on their brain development. Critical brain development was the age when important brain growth was still ongoing, synapses maturing, and cells developing. Low brain development was categorized when animals reached puberty/ adolescence. Ongoing brain development the time in between. For rats and mice the following groups were determined: critical brain development (0–20 postnatal days)^43,44^, ongoing brain development (21–42 postnatal days)^45^ and low brain development (43–60 postnatal days)^46^. For the rhesus macaque monkeys the same categories were defined with the following ages: critical brain development (0–5 months)^47,48^, ongoing brain development (5 months–2.5 years) and low brain development (2.5–5 years)^39^.

The effect of the timing of the outcome assessment was assessed in 2 groups. Short term effects were effects measured within 1 month (30 days) after exposure, and long term effects after 1 month of exposure^49,50^.

The results of subgroup analyses were only interpreted when subgroups contained at least data from ten independent studies.

We expected the variance to be comparable within the subgroups; therefore, we assumed a common among-study variance across subgroups. For subgroup analyses, we adjusted our significance level according to the conservative Bonferroni method to account for multiple analyses (p* number of comparisons). Results are considered statistically significant when P is lower or equal to 0.05. However, differences between subgroups should be interpreted with caution and should only be used for constructing new hypotheses rather than for drawing final conclusions.

### Sensitivity analyses

In order to assess the robustness of the results, sensitivity analyses were planned. We assessed the effect (1) not normally distributed outcomes (e.g. medians), (2) studies in which the set age was either 42^36^ or 21^51^ days instead of 60 days (3) studies with exposures longer than 3 h, (4) changing the limits for short and long term effects (e.g. short term will be divided into short term of 0 to 24 h and moderate term of 1 to 30 days, long term remains > 30 days) (5) and in- or excluding extreme outliers.

### Risk of bias

We used the SYRCLE Risk of Bias tool^52^ to assess the risk of bias in 50 studies (n = 20 isoflurane, n = 20 sevoflurane and n = 10 desflurane). For isoflurane and sevoflurane the references assessed were randomly selected. For enflurane all included studies in the systematic review were assessed.

Two independent reviewers assessed the risk of bias in the included paper (MB, TS, NK).

A ‘yes’ score indicates low risk of bias; a ‘no’ score indicates high risk of bias; and a ‘?’ score indicates unknown risk of bias.

To overcome the problem of judging too many items as “unclear risk of bias” because reporting of experimental details on animals, methods and materials is generally very poor^53,54^ we added two items on reporting: reporting of any measure of randomization, reporting of any measure of blinding. For these two items, a ‘yes’ score indicates ‘reported’, and a ‘no’ score indicates ‘unreported’.

### Publication bias

In case of 20 or more independent studies, we used funnel plots and Trim and Fill analysis to search for evidence of publication bias. Because SMDs may cause funnelplot distortion we plotted the SMD against a sample size-based precision estimate (1/√(n))^55^.

## Results

### Adjustments to the protocol

Before the start of data-extraction, it was decided to change the minimal amount of studies to perform subgroup analyses from three to ten studies per outcome. For publication bias, the minimal amount of comparisons was changed from fifteen to twenty per outcome. These changes were made because both the results of subgroup analyses and publication bias analyses in case of heterogenous animal studies appear to be more robust and valuable when more studies are included.

### Study selection

**Figure 1:**
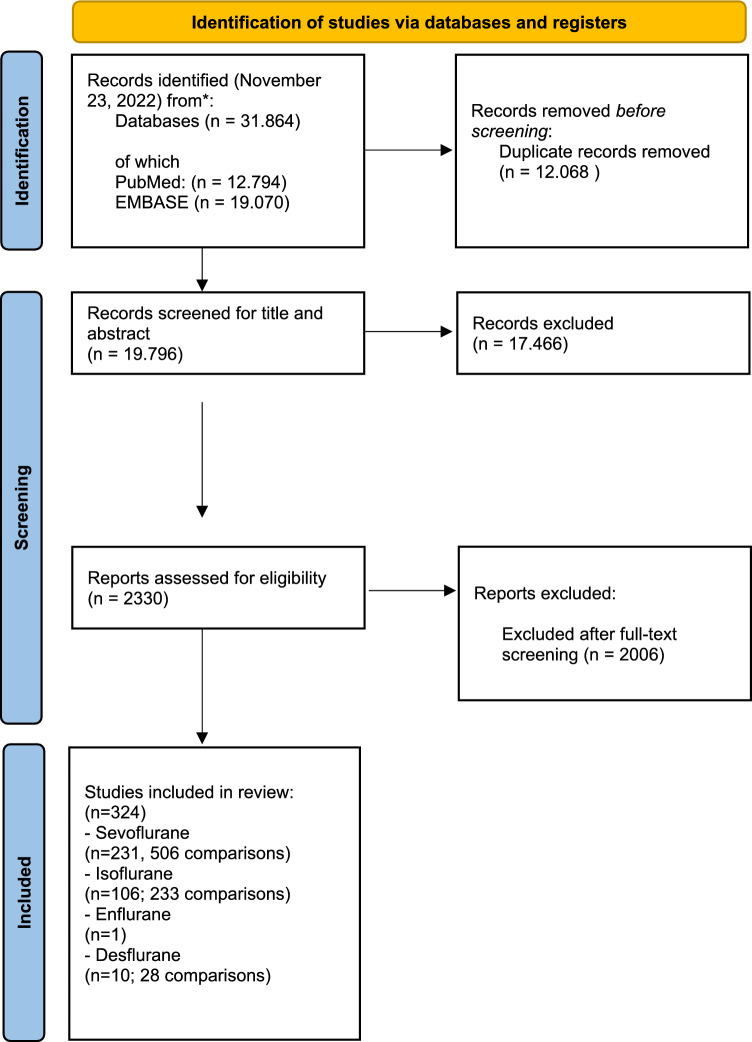
shows the flow chart of our study selection process. From^32^: For more information, visit: http://www.prisma-statement.org/.

Figure 1 shows the flow chart of our study selection process. Over 30.000 abstracts were identified, and after deduplication 19.796 abstracts were screened by 2 independent reviewers. 231 references could be included investigating the effects of sevoflurane on neurodegeneration or behaviour in young animals, n = 106 about isoflurane, n = 10 about desflurane, and n = 1 studies regarding enflurane. The reference list of all included studies (n = 324) can be found in supplemental file 3.

### Study characteristics

#### Sevoflurane

**Figure 2:**
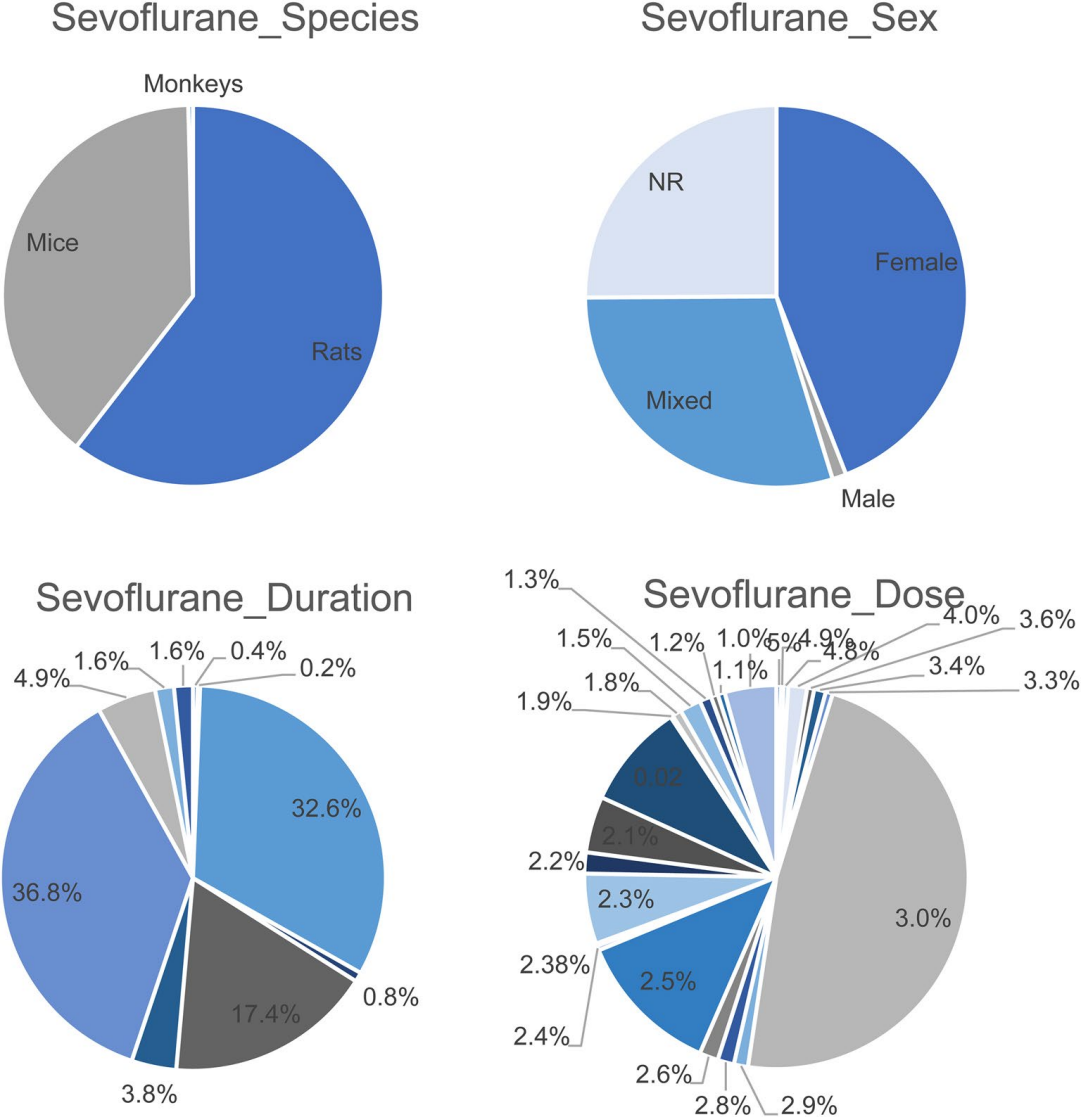
Summary of the Sevoflurane study characteristics species, sex, dose and duration of exposure.

All characteristics regarding sevoflurane exposure are summarized in supplemental file 4 and Fig. 2.

233 studies containing n = 506 comparisons were included in this analysis. 60.5% of the comparisons used rats. Mice and monkeys were used in 39.1% and 0.4% of the comparisons, respectively. Most of the animals that were used for the experiments, were either males (n = 223, 44.1%), or both sexes (n = 150, 29.6%). Few comparisons used female animals (n = 6, 1.2%). 127 comparisons did not report the sex of the animals that were used (25.1%). In almost all of the cases, the animals received the sevoflurane as an inhalational agent (n = 504, 99.6%). Only one comparison administered sevoflurane intraperitoneally, one other comparison did not report the administration route of sevoflurane. In the majority of the comparisons (93.5%, n = 473), animals were exposed to sevoflurane during the critical brain development phase (postnatal day 0–20), of which 89.9% (n = 423) was on postnatal day 6 or 7. In 4% (n = 20) and 2.2% (n = 11) of the comparisons, animals were exposed in the ongoing brain development phase and low brain development phase. In two comparisons (0.4%) it was unclear when the animals were exposed to sevoflurane. The doses of sevoflurane that were used the most for administration in these animals, were respectively 3% (n = 241), 2.5% (n = 62), 2.0% (n = 45), 2.3% (n = 30) and 2.1% (n = 24). The animals were most often exposed to sevoflurane for a duration of 2 h (n = 186), followed by 6 h (n = 165), 4 h (n = 88), 1 h (n = 25) and 3 h (n = 19). The most common neurodegenerative outcomes used were: caspase-3, followed by TUNEL, Bax, cleaved-PARP and apoptotic cell number (respectively n = 236, n = 46, n = 27, n = 14 and n = 10 comparisons). Regarding the outcomes assessing behavioral changes, the most common outcomes used were: MWM, Open Field test, Fear conditioning, Elevated Plus Maze and Novel object recognition (respectively n = 203, n = 79, n = 58, n = 28, n = 20 comparisons). In 44.0% (n = 107) of the comparisons assessing a neurodegenerative outcome, the outcome was measured directly after the exposure to sevoflurane. Other frequent timing of outcome measures were 2 h after exposure (5.3%; n = 13), 6 h after exposure (16.0%; n = 39), 12 h after exposure (3.7%; n = 9) and 18 h after exposure to sevoflurane (4.1%; n = 10). Five comparisons did not mention the timing of outcome measure. The timing of outcome measure of the behavioral test is more scattered. In 7.3% (n = 40) of the comparisons assessing a behavioral outcome, the outcome was measured 28 days after the exposure to sevoflurane. The other most frequent timing of outcome measures were 21 days after exposure (5.3%; n = 29), 49 days after exposure (4.2%; n = 23) and 23 days after exposure to sevoflurane (3.8%; n = 21). Seventeen comparisons did not mention the timing of outcome measure.

#### Isoflurane

**Figure 3:**
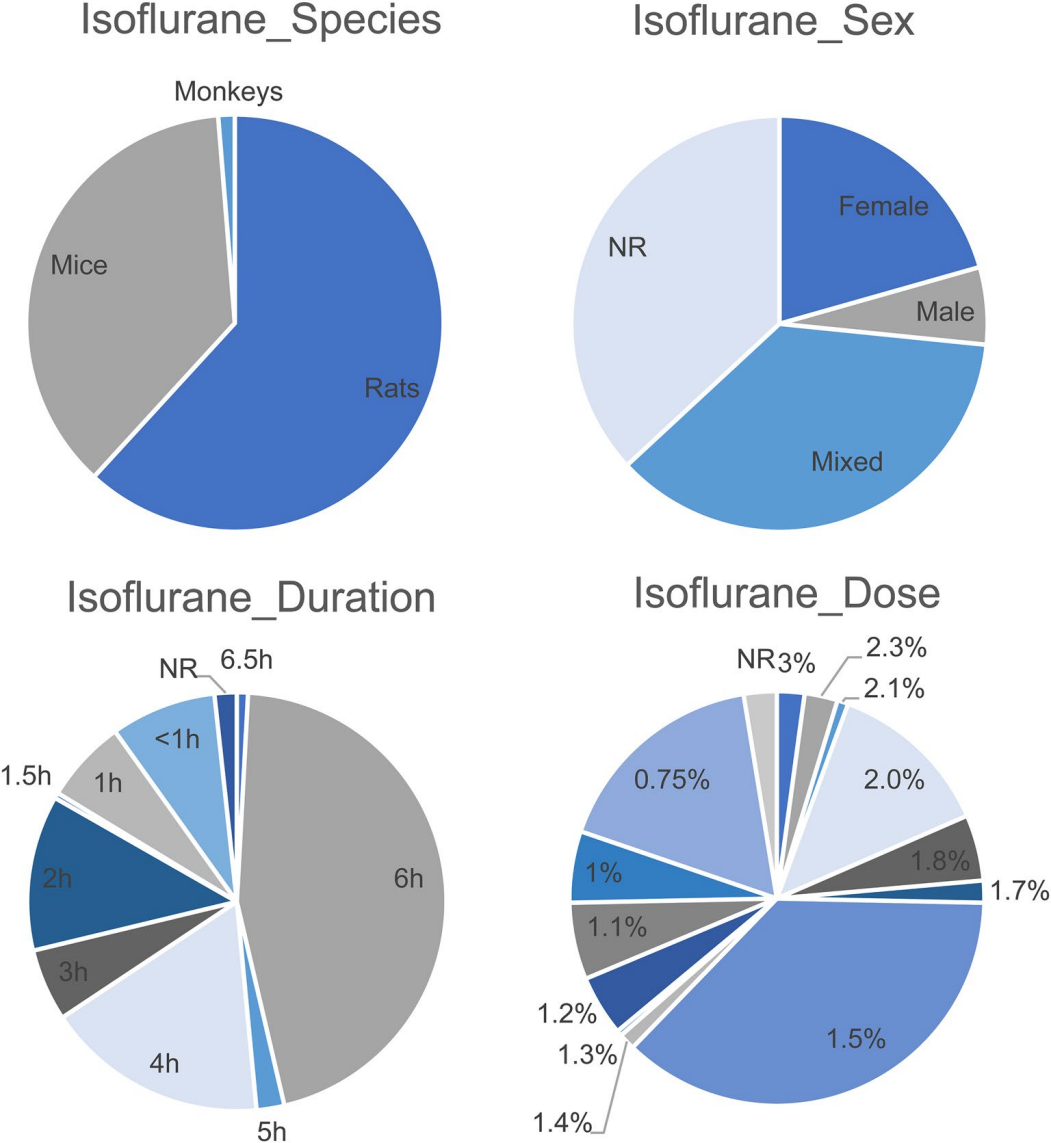
Summary of the Isoflurane study characteristics species, sex, dose and duration of exposure.

All characteristics regarding isoflurane exposure are summarized in supplemental file 5 and Fig. 3.

106 references, including 233 individual comparisons could be included in this SR about the effects of isoflurane in the young animals. From these comparisons, 61.8% (n = 144) used rats, 36.9% (n = 86) used mice and 1.3% monkeys (n = 3). Most studies used mixed sex groups (n = 85, 36.5%), while 20.6% (n = 48) used only males and 6.0% (n = 14) used female animals The remaining 36.9% (n = 86) of the comparisons failed to report the sex of the animals.

95.3% (n = 222) of the comparisons exposed the animals to isoflurane during the critical brain development phase, of which 79.8% (n = 186) was on postnatal day 6 or 7. The other 2.1% (n = 5), 1.3% (n = 3) and 1.3% (n = 3) respectively, exposed the animals during the ongoing brain development phase, low brain development and brain development phase was not reported.

Most studies used 1.5% isoflurane (36.9%; n = 86) and the exposure lasted for 6 h in 47.5% (n = 106). Caspase-3 was the most frequently assessed neurodegenerative outcome (n = 120, 51.5%). Other frequently assessed neurodegenerative outcomes were; TUNEL (n = 71, 30.5%), Bax (n = 16, 6.9%), tAIF (n = 12, 5.2%), PARP (n = 11, 4.7%) and Fluoro Jade (n = 10, 4.3%).

The most frequently assessed behavioral tests were the Morris water maze (n = 69, 29.6%), open field test (n = 31, 13.3%), novel object recognition test (n = 19, 8.2%), elevated plus maze (n = 18, 7.7%), fear conditioning (n = 15, 6.4%) radial arm maze (n = 14, 6.0%), locomotor activity by rotarod (n = 14, 6.0%), social recognition (n = 13, 5.6%) and locomotor activity (n = 10, 4.4%). Most comparisons assessed the outcomes relatively short after exposure. Regarding the neurodegenerative outcomes, 93.3% measured effects before 30 days and 6.7% (n = 8) did not report timing outcome.

Concerning the behavioural outcome assessment, the majority of the comparisons (49.6%; n = 56) assessed the effect of isoflurane exposure after 30 days, 43.3% measured outcome assessment before 30 days and 7.0% (n = 8) did not report timing of outcome assessment. Overall, 69.1% (n = 161) outcomes measured effects before 30 days (short term), 24.0% (n = 56) measured effects after 30 days (long term) and 6.9% (n = 16) did not report timing of outcome assessment.

#### Desflurane

**Figure 4:**
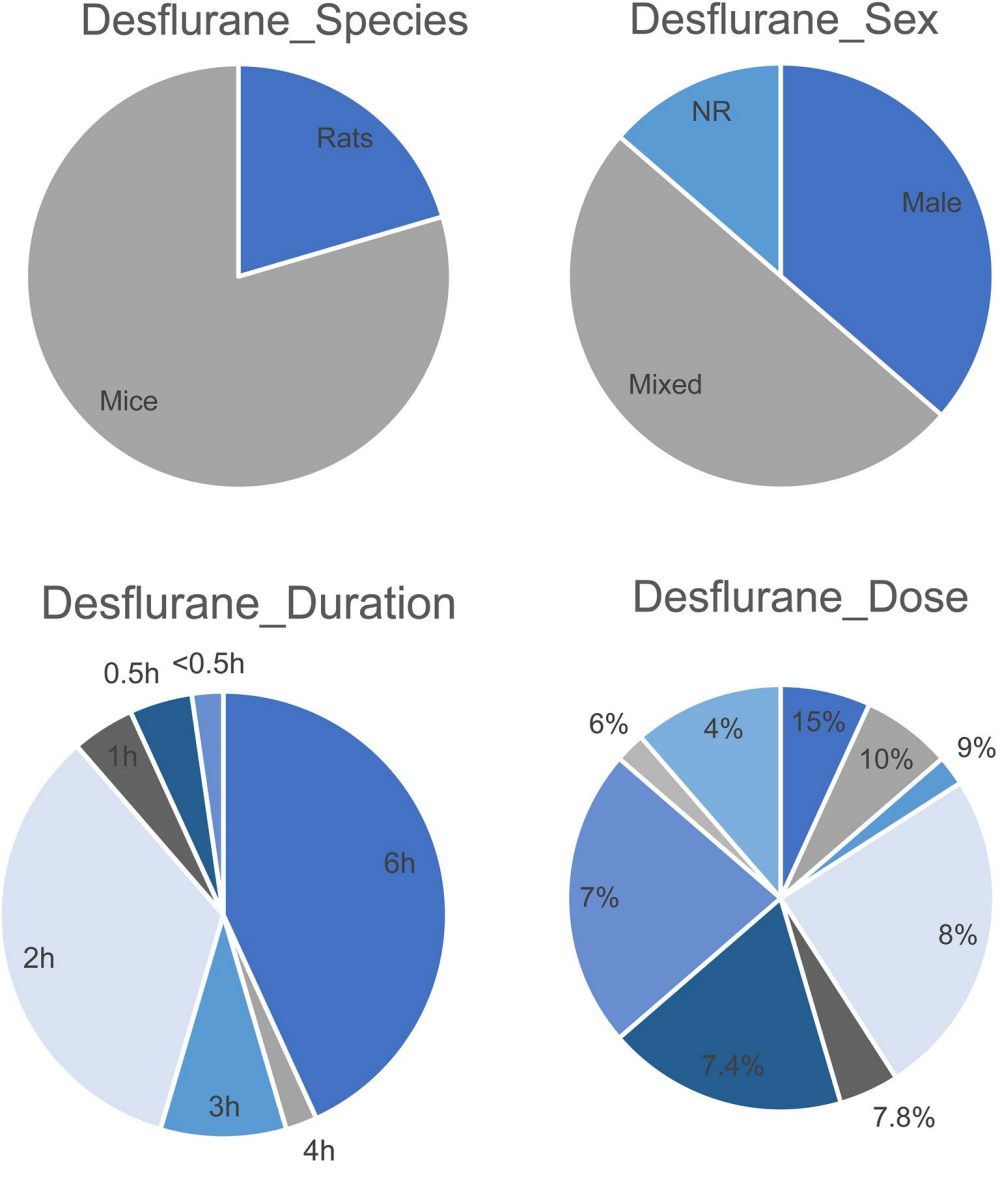
Summary of the Desflurane study characteristics species, sex, dose and duration of exposure.

All characteristics regarding desflurane exposure are summarized in supplemental file 6 and Fig. 4.

Ten studies containing 44 comparisons were included in this systematic review. All of the comparisons used either mice or rats (respectively 79.5%; n = 35 and 20.5%; n = 9).

In 36.4% (n = 16) of the comparisons studied males, and 50.0% (n = 22) mixed sex groups. The remaining comparisons did not mention the sex of the used animals (13.6%; n = 6). There were no comparisons using solely females. Like for isoflurane and sevoflurane the majority of the studies were exposed to desflurane during the critical development phase (95.5%, n = 42), of which 76.2% (n = 32) was on either postnatal day 6 or 7. The remaining 4.5% (n = 2) was exposed during the low brain development phase. In all individual comparisons, desflurane was administered via inhalation. The analysis shows that 63.6% (n = 28) of the comparisons used a dose of desflurane between 7.0- and 8.0%. Animals were exposed to a single dose of desflurane in 90.9% (n = 40) of the comparisons. In four comparisons (9.1%), animals were exposed to multiple doses of desflurane. The majority of comparisons studies exposed the animals to desflurane for either 6 (n = 19), 3 (n = 4) or 2 (n = 12) h. Twenty-one of the included comparisons assessed a neurodegenerative outcome, twenty-three comparisons assessed a behavioral outcome. Caspase-3 was the most popular neurodegenerative outcome (38.1%; n = 8) TUNEL assay was used in 28.6% (n = 6) of the comparisons. In 30.4% (n = 7) of the comparisons the Morris water maze was used to assess learning or memory. The Fear Conditioning Test was conducted in 21.7% (n = 5) of the comparisons, the open-field test in 17.4% (n = 4) of the comparisons and the elevated plus maze in 8.7% (n = 2) of the comparisons. In 57.1% (n = 12) of the comparisons assessing a neurodegenerative outcome, the outcome was measured directly after the exposure to desflurane. In 42.8% (n = 9) the timing of outcome assessment was between 4 and 6 h after desflurane exposure. Behavioral assessment were conducted much later. In 30.4% (n = 7) of the comparisons assessing a behavioral outcome, the outcome was measured 22 days after the exposure to desflurane. In respectively 17.4% (n = 4) and 13.0% (n = 3) of the comparisons behavioral outcome assessment was either 28 days or 36 days after the exposure to desflurane.

#### Enflurane

Regarding the exposure to enflurane, only 1 manuscript was identified containing 4 comparisons (supplemental file 7). This study was conducted in rabbits, and did not study any of our predefined outcomes for meta-analysis.

### Risk of bias

Risk of bias was assessed in 50 independent studies (n = 20 isoflurane, n = 20 sevoflurane and n = 10 desflurane). For isoflurane and sevoflurane the references assessed were randomly selected.

The results of the risk of bias analyses are shown in supplemental file 8.

Supplemental file 8 clearly shows that the majority of the risk of bias questions assessed scored an unclear risk of bias, which is due to poor reporting of essential methodological details. For example none of the included studies in the risk of bias analysis provided sufficient details to assess (1) whether or not the animal were selected at random during the outcome assessment, (2) the allocation to the different groups was adequately concealed and (3) the animals were randomly housed during the conduct of the study. In addition, there was only 1 out of the 50 studies that clearly described how they generated and applied the allocation sequence?

Because of the poor reporting of essential details we added to reporting quality questions. 80% of the studies described that they randomised at least at one level (generally during the selection of the animals across the groups). Almost 50% reported that they blinded the study at least at one level (generally during outcome assessment).

### Meta analysis

For each halogenated ether neurodegenerative (caspase-3 and Tunel), anxiety (open field and Elevated plus maze) and learning and memory (Morris water maze, contextual fear conditioning, cued fear conditioning) related outcomes were quantitively analysed. The extracted data and individual effectsizes can be found in supplemental file 9.

#### Sevoflurane

##### Caspase-3

**Figure 5:**
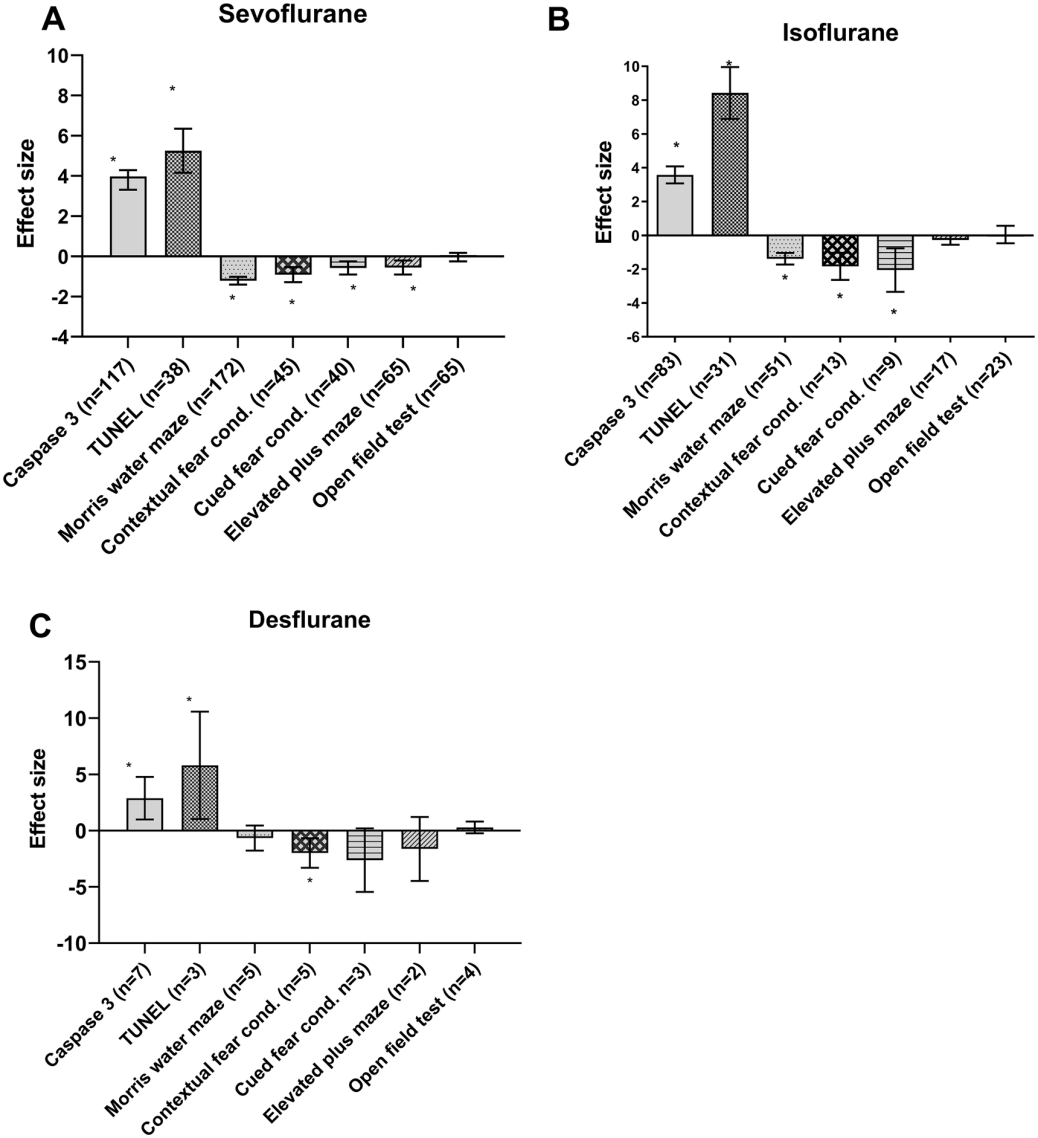
Results of the overall analyses regarding the exposure to sevoflurane, isoflurane and desflurane. (**A**): overall analyses sevoflurane, (**B**): overall analyses isoflurane, (**C**): overall analyses desflurane. The columns indicate the overall effect estimate with the 95% confidence interval of the various outcomes investigated in this SR. * represents a significant effect (*p* < 0.05)

**Figure 6:**
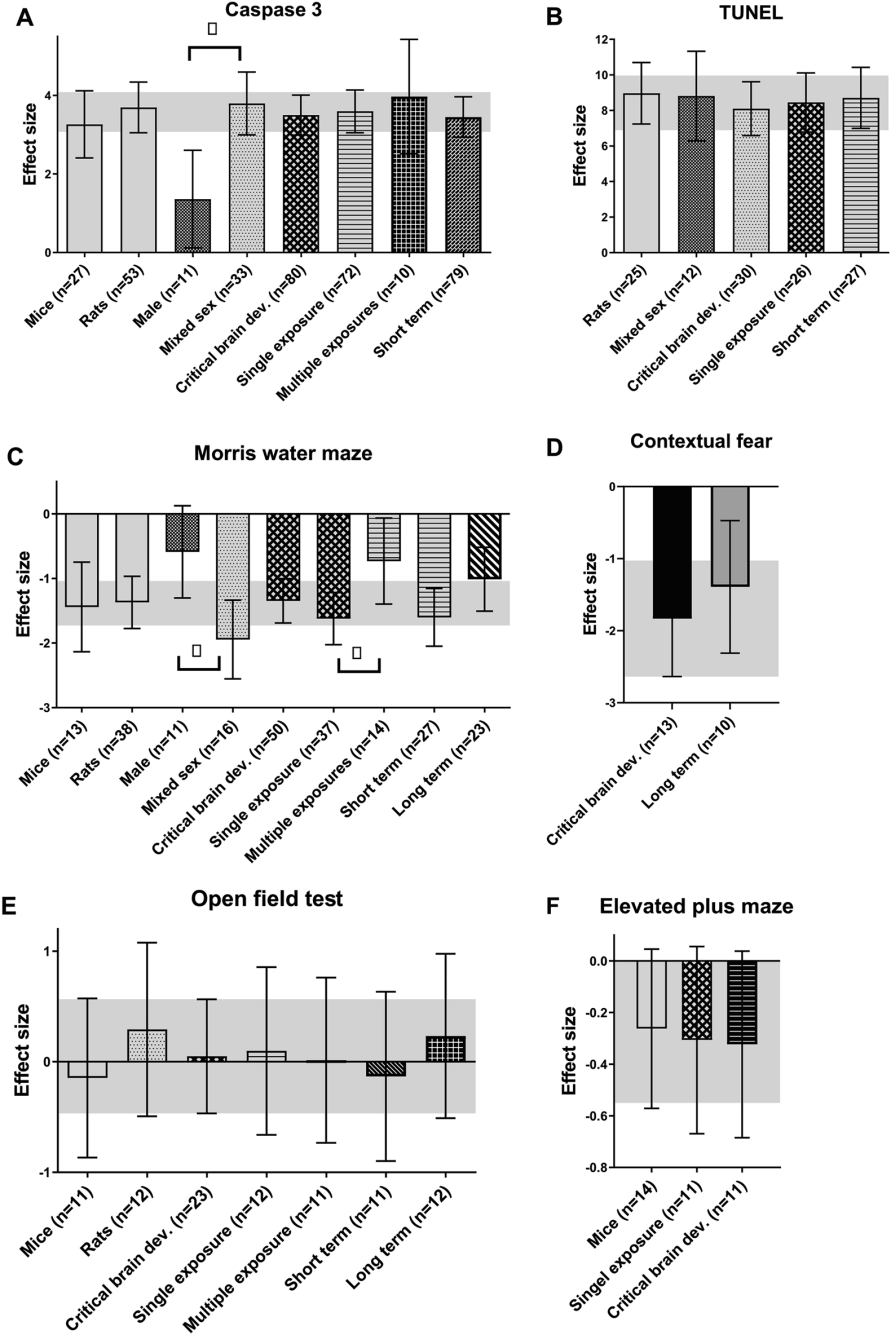
Results of the subgroup analyses regarding the exposure to sevoflurane. (**A**) Caspase-3, (**B**) Tunel, (**C**) Morris water Maze, (**D**) Contextual fear conditioning, (**E**) Cued fear conditioning, (**F**) Elevated plus maze, (**G**) Open field test. The grey bars represent the 95% confidence interval of the pooled effect estimate (hedges **G**). The columns indicate the effect estimate (Hedges **G**) with the 95% confidence interval of the subgroups. The results from subgroup analyses were only displayed when subgroups contained data of at least 10 independent comparisons.

Seventy-four different studies containing 153 comparisons were extracted, of which 117 independent comparisons were included in the meta-analysis. Figure 5A shows that exposure to sevoflurane significantly increases Caspase-3 levels (Hedges g 3.797 [3.308; 4.286], n = 117, I^2^ = 85.3%). Subgroup analysis revealed no significant differences between subgroups. It should be noted however that many subgroups contained too few comparisons for reliable analyses. (rats n = 77, mice n = 39, monkeys n = 1, males n = 31, females n = 0 and the mixed sex groups n = 52, n = 34 did not report the sex of the animals, critical brain development n = 113, ongoing brain development n = 3, low brain development n = 1, no details about the brain development phase n = 0, single exposure n = 101, multiple exposures n = 16, timing outcome assessment relative to exposure short term n = 113, long term n = 3, unclear n = 1). The results of the subgroups that could be analysed or compared are shown in Fig. 6A.

##### Tunel

Thirty-two different studies containing 46 comparisons were extracted, of which 38 independent comparisons were included in the meta-analysis. As shown in Fig. 5A inhalation of sevoflurane increases levels of TUNEL significantly (Hedges g 5.253 [4.156; 6.350], n = 38, I^2^ = 90.3%).

Subgroup analyses revealed no significant differences between subgroups. It should be noted however that many subgroups contained too few comparisons for reliable analyses. (rats n = 20, mice n = 18, Mixed sex n = 12, males n = 11, and n = 15 did not report the sex of the animals, critical brain development n = 36, ongoing brain development n = 1 and no details about the brain development phase n = 1, single exposure n = 26, multiple exposures n = 12, timing outcome assessment relative to exposure short term n = 36, long term n = 1, unclear n = 1). The results of the subgroups that could be analysed are shown in Fig. 6B.

##### MWM

**Figure 7:**
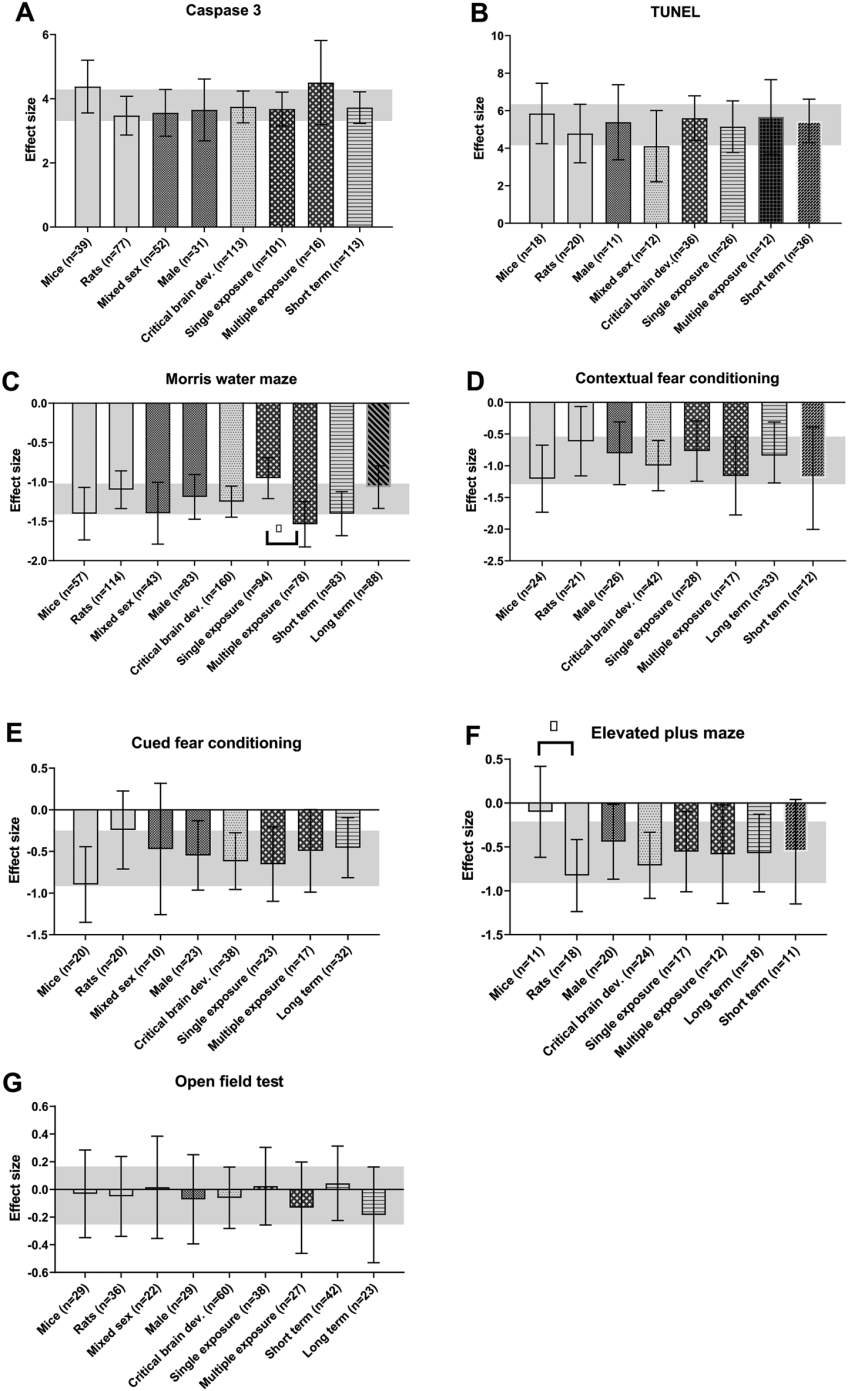
Results of the subgroup analyses regarding the exposure to isoflurane. (**A**) Caspase-3, (**B**) Tunel, (**C**) Morris water Maze, (**D**) Contextual fear conditioning, (**E**) Cued fear conditioning, (**F**) Elevated plus maze, (**G**) Open field test. Open field test. The grey bars represent the 95% confidence interval of the pooled effect estimate (hedges **G**). The columns indicate the effect estimate (Hedges **G**) with the 95% confidence interval of the subgroups. The results from subgroup analyses were only displayed when subgroups contained data of at least 10 independent comparisons

One hundred and sixteen different studies containing 219 comparisons were extracted, of which 172 independent comparisons were included in the meta-analysis. As shown in Fig. 5A, inhalation of sevoflurane decreases the time spent in the target quadrant and the platform crossings in the MWM significantly (Hedges g − 1.217 [− 1.411; − 1.023], n = 172, I^2^ = 82.3%). Subgroup analysis for exposure revealed that the time spent in the target quadrant is significantly less (*p* = 0.005) in animals that were multiple times exposed to sevoflurane (Hedges g − 1.538 [− 1.825; − 1.250]; n = 78; I^2^ = 87.2%) compared to animals exposed only one time (Hedges g − 0.952 [− 1.211; − 0.694]; n = 94; I^2^ = 72.3%) (Figure Fig. 7c). Other subgroup analyses revealed no significant differences between subgroups, or groups were too small to reliably estimate the subgroup effects (mice n = 57, rats n = 114, monkeys n = 1, mixed sex groups n = 43, males n = 83, females = 3, not reported n = 43, critical brain development n = 160, ongoing brain development n = 2, low brain development n = 9 and no details about the brain development phase n = 1, single exposure n = 94, multiple exposures n = 78, no details about exposure n = 0, timing outcome assessment relative to exposure short term n = 83, long term n = 88, unclear n = 1). The results of the subgroups that could be analysed are shown in Fig. 6C.

##### Contextual fear conditioning

Thirty-eight different studies containing 45 independent comparisons were included in the meta-analysis. As shown in Fig. 5A, inhalation of sevoflurane decreases the freezing response in the contextual Fear Conditioning Test significantly (Hedges g − 0.915 [− 1.291; − 0.538]), I^2^ = 85.4%).

Comparisons between subgroups could be conducted for species,exposure and timing outcome assessment. However, no differences between mice n = 24 and rats n = 21, single n = 28 versus multiple exposure n = 17, and long term n = 33 versus short term n = 12 were observed. All other subgroups were either too small or only one group was available for analysis (mixed sex groups n = 8, males n = 26, females = 0, not reported n = 11, critical brain development n = 42, ongoing brain development n = 2, low brain development n = 1 and no details about the brain development phase n = 0. The results of the subgroups that could be analysed are shown in Fig. 6D.

##### Cued fear conditioning

Thirty individual studies containing 40 independent comparisons were included in the meta-analysis. As shown in Fig. 5A, exposure to sevoflurane decreases the freezing response in the cued Fear Conditioning Test significantly (Hedges g − 0.581 [− 0.910; − 0.251], n = 40, I^2^ = 75.6%). Predefined subgroup analyses were performed to explore the heterogeneity.

Comparisons between subgroups could be conducted for species, sex and exposure Fig. 6E. No significant differences were observed between mice (n = 20) and rats (n = 20) and males (n = 23) and mixed sex (n = 10) groups or single n = 23 versus multiple exposure n = 17 were observed. All other subgroups were either too small or only one group was available for analysis (females = 0, not reported n = 7, critical brain development n = 38, ongoing brain development n = 2, no details about the brain development phase n = 0, timing outcome assessment relative to exposure short term n = 8, long term n = 32, unclear n = 0).

##### Elevated plus maze

Nineteen individual studies containing 29 independent comparisons were extracted and included in the meta-analysis. As shown in Fig. 5A, exposure to sevoflurane significantly decreases the time spent in the open arm in the Elevated Plus Maze (Hedges g − 0.562 [− 0.911; − 0.213], n = 29, I^2^ = 74.5%). Predefined subgroup analyses were performed to explore the heterogeneity (Fig. 6F). Comparisons between subgroups could be conducted for species, exposure and timing outcome assessment. Rats (Hedges g − 0.826 [− 1.237; − 0.416]; n = 18; I^2^ = 69.6%) spent significantly (*p* = 0.04) less time in the open arm in the elevated plus maze compared to mice (Hedges g − 0.100 [− 0.619; 0.418]; n = 11; I^2^ = 70.0%). However, no differences between single n = 17 versus multiple exposure n = 12 and long term n = 18 and short term n = 11 were observed. All other subgroups were either too small or only one group was available for analysis (mixed sex groups n = 3, males n = 20, females = 2, not reported n = 4, critical brain development n = 24, ongoing brain development n = 5, low brain development n = 0 and no details about the brain development phase n = 0.

##### Open field test

Fifty-two individual studies containing 88 comparisons were extracted, of which 65 independent comparisons were included in the meta-analysis (Fig. 5A). Exposure to sevoflurane has no significant effect on the total distance traveled in the Open Field Test (Hedges − 0.042 [− 0.254; 0.169], n = 65, I^2^ = 71.7%).

Comparisons between subgroups could be conducted for species, sex, exposure and timing outcome assessment. However, no significant differences between any of these groups was observed (Fig. 6G). All other subgroups were either too small or only one group was available for analysis (females = 1, ongoing brain development n = 5, low brain development n = 0 and no details about the brain development phase n = 0,

#### Isoflurane

##### Caspase-3

Sixty individual studies containing 108 comparisons investigating the effect of isoflurane on caspase-3 levels were extracted, of which 89 independent comparisons could be included in meta-analysis. Figure 5B shows that Isoflurane exposure significantly increased caspase-3 levels compared to control exposed animals (Hedges g = 3.580 [3.075; 4.084]; n = 83 I^2^ = 87.05%). Subgroup analyses revealed that experimental groups of mixed sex (Hedges G 3.795 [2.995; 4.595]; n = 33; I^2^ = 89.1%) showed significantly (*p* = 0.002) higher caspase-3 levels compared to male animals (Hedges g 1.360 [0.114; 2.605]; n = 11; I^2^ = 56.0%). No significant differences could be observed between rats n = 53, mice n = 27 and monkeys n = 3 or single exposure n = 72 and multiple exposures n = 10. It should be noted however that many subgroups contained too few comparisons for reliable analyses. (critical brain development n = 80, ongoing brain development n = 1, low brain development n = 1 and no details about the brain development phase n = 1, timing outcome assessment relative to exposure short term n = 79, long term n = 0, unclear n = 1). The results of the subgroups that could be analysed are shown in Fig. 7A.

##### Tunel

Thirty individual studies containing 40 comparisons were extracted, of which 31 individual comparisons could be included in meta-analysis (Fig. 5B). The Tunel assay showed significantly more apoptosis in isoflurane exposed animals compared to control exposed animals hedge’s g = 8.425 [6.888; 9.961] n = 31 I^2^ = 89.2).

Subgroup analyses revealed no significant differences between subgroups. It should be noted however that many subgroups contained too few comparisons for reliable analyses (rats n = 25, mice n = 6, Mixed sex n = 12, males n = 3, and n = 16 did not report the sex of the animals, critical brain development n = 30, and no details about the brain development phase n = 1, single exposure n = 26, multiple exposures n = 4, no details about exposure n = 1, timing outcome assessment relative to exposure short term n = 27, long term n = 0, unclear n = 4). The results of the subgroups that were large enough are shown in Fig. 7B.

##### Morris water maze

Fifty individual studies containing 68 comparisons were extracted, of which 51 individual comparisons could be included in meta-analysis. The overall analysis showed significant learning and memory deficits in isoflurane exposed animals (Hedges g; − 1.384 [− 1.728; − 1.040] n = 51 I^2^ = 79.9) (Fig. 5B). Subgroup analyses revealed that this effect is larger (*p* = 0.007) in the mixed sex group (Hedges g − 1.946 [-2.555; − 1.337]; n = 16; I^2^ = 83.5%) compared to the male subgroup (Hedges g -0.590 [− 1.303; 0.124]; n = 11; I^2^ = 75.9%). In addition multiple exposures to isoflurane (Hedges g -0.733 [− 1.399; − 0.066]; n = 14; I^2^ = 78.8%) appeared to impair learning and memory significantly more (p = 0.03) compared to single exposure to isoflurane (Hedges g − 1.623 [− 2.026; − 1.220]; n = 37; I^2^ = 80.4%) after.

Other subgroup analyses revealed no significant differences between subgroups, or groups were too small to reliably estimate the subgroup effects rats n = 38, mice n = 13, critical brain development n = 50, and no details about the brain development phase n = 1, long term n = 23, unclear n = 1 were observed. The results of the subgroups that were large enough are shown in Fig. 7C.

##### Contextual fear conditioning

The effects of isoflurane exposure on learning and memory deficits was also assessed using the contextual fear conditioning test. Thirteen individual comparisons could be included in meta-analysis.

Isoflurane significantly decreased the freezing responses in isoflurane exposed animals, indicating impaired learning and memory (Hedges g − 1.832 [− 2.637; − 1.027] n = 13, I^2^ = 86.0) (Fig. 5B). No subgroup analyses could be conducted, as all subgroups contained too few comparisons for reliable analyses (rats n = 4, mice n = 9, Mixed sex n = 4, males n = 5, females n = 2, and n = 2 did not report the sex of the animals, critical brain development n = 13, single exposure n = 7, multiple exposures n = 6, timing outcome assessment relative to exposure short term n = 3, long term n = 10.

##### Cued fear conditioning

Nine individual comparisons could be included in meta-analysis of the cued fear conditioning test. Isoflurane significantly decreased freezing response compared to the control exposed animals (Hedges g − 2.057 [− 3.345; − 0.770] n = 9, I^2^ = 90.0) Fig. 7D. Also for the cued fear conditioning all subgroups were too small for reliable subgroup analyses (rats n = 4, mice n = 5, Mixed sex n = 3, males n = 4, females n = 1, and n = 1 did not report the sex of the animals, critical brain development n = 9, single exposure n = 5, multiple exposures n = 4, timing outcome assessment relative to exposure short term n = 3, long term n = 6).

##### Elevated plus maze

Nine individual studies containing 22 comparisons were extracted, of which17 comparisons could be included in meta analysis. Isoflurane tends to reduce the time spent in the open arm (Hedge’s g = − 0.275 [− 0.551; 0.000] n = 17, I2 = 28.7), indicating a small difference in anxiety between isoflurane and control exposed animals Fig. 5B. No comparsons between subgroups could be made due to too few comparsons in most subgroups (rats n = 3, mice n = 14, Mixed sex n = 7, males n = 5, females n = 2, and n = 3 did not report the sex of the animals, critical brain development n = 11, ongoing brain development n = 4, low brain development n = 2, single exposure n = 11, multiple exposures n = 6, timing outcome assessment relative to exposure short term n = 5, long term n = 5 and n = 7 did not report timing outcome assessment).

#### Open field test

Twenty-one individual studies containing 31 comparisons were extracted, of which 23 individual comparisons could be included in meta-analysis. Isoflurane did not alter anxiety related behavior in the open field tests (hedge’s g = 0.049 [− 0.467; 0.565] n = 23, I^2^ = 81.4) Fig. 5B. Subgroup analysis showed no significant difference between subgroups. The groups that contained enough comparisons are depicted in Fig. 7E. The number of animals per subgroup were respectively; rats n = 12, mice n = 11, Mixed sex n = 4, males n = 9, females n = 4, and n = 6 did not report the sex of the animals, critical brain development n = 23, single exposure n = 12, multiple exposures n = 11, timing outcome assessment relative to exposure short term n = 11, long term n = 12.

#### Desflurane

##### Caspase-3

Four individual studies containing 8 comparisons were extracted, of which 7 comparisons could be included in the meta-analysis (Fig. 5C). Exposure to desflurane significantly increases the levels of Caspase-3 (Hedges g 2.883 [0.996; 4.770], n = 7, I^2^ = 89.9%). No subgroup analyses could be performed due to limited number of studies (rats n = 1, mice n = 6, mixed sex n = 3, males n = 4, critical brain development n = 7, single exposure n = 7, timing outcome assessment relative to exposure short term n = 7).

Tunel.

Three individual studies containing 6 comparisons were extracted, of which only 3 comparisons could be included in meta-analysis (Fig. 5C). The Tunel assay showed significantly more apoptosis in isoflurane exposed animals compared to control exposed animals hedge’s g = 5.80 [1.03; 10.58] n = 3 I^2^ = 92%) (mice n = 3, mixed sex n = 1, males n = 2, critical brain development n = 3, single exposure n = 3, timing outcome assessment relative to exposure short term n = 3).

##### MWM

Five individual studies containing five comparisons were extracted and could be included in the meta-analysis (Fig. 5C). Exposure to desflurane did not cause significant learning and memory deficits that could be observed with the Morris water maze test (Hedges g 20.67 [0.781; 4.450], n = 5, I^2^ = 82.9%). No subgroup analyses could be performed due to limited number of studies (rats n = 1, mice n = 4, mixed sex n = 3, males n = 2, critical brain development n = 3, low brain development n = 2, single exposure n = 3, multiple exposure n = 2, timing outcome assessment relative to exposure short term n = 4, long term n = 1).

##### Contextual Fear conditioning

Exposure to desflurane (Fig. 5C) decreases the freezing response in the contextual fear conditioning test significantly (Hedges g − 1.990 [− 3.304; − 0.676], n = 5, I^2^ = 85.9%).

No subgroup analyses could be performed due to limited number of studies (mice n = 5, mixed sex n = 4, males n = 1, critical brain development n = 5, single exposure n = 4, multiple exposures n = 1, timing outcome assessment relative to exposure short term n = 3, long term n = 2).

##### Cued fear conditioning

Three comparisons were included in the meta-analysis. As shown in Fig. 2C, exposure to desflurane did not alter the freezing responses in the cued fear conditioning test (Hedges g − 2.624 [− 5.442; 0.194] n = 3, I^2^ = 93.5%).

No subgroup analyses could be performed due to limited number of studies (mice n = 3, mixed sex n = 2, males n = 1, critical brain development n = 3, single exposure n = 2, multiple exposures n = 1, timing outcome assessment relative to exposure short term n = 1, long term n = 2).

##### Elevated plus maze

Two individual studies containing 2 comparisons were extracted and could be included in the meta-analysis assessing the effect of desflurane in the elevated plus maze. Figure 5C shows that exposure to desflurane did not significantly alter the time spent in the open arm (Hedges g − 1.627 [− 4.469; 1.215], n = 2, I^2^ = 90.4%.

No subgroup analyses could be performed due to limited number of studies (mice n = 2, mixed sex n = 1, males n = 1, critical brain development n = 2, single exposure n = 2, timing outcome assessment relative to exposure short term n = 1, long term n = 1).

##### Open field test

Three individual studies containing 4 comparisons were extracted and could be included in the meta-analysis assessing anxiety related behavior. Figure 5C shows that exposure to desflurane did not alter the total distance traveled (Hg 0.281 [− 0.239; 0.801], n = 4, 47.1%) compared to control exposed animals.

No subgroup analyses could be performed due to limited number of studies (mice n = 4, mixed sex n = 3, males n = 1, critical brain development n = 4, single exposure n = 4, timing outcome assessment relative to exposure short term n = 3, long term n = 1).

#### Summary of the effects of all meta analyses

Table 1 summarizes the direction of effects of all conducted meta analyses.

**Table 1:** Summary of the direction of effects of all overall meta analyses per flurane.

	Sevoflurane	Isoflurane	Desflurane
ND: Caspase-3	↑ (n = 117)	↑ (n = 89)	↑ (n = 7)
ND: TUNEL	↑ (n = 38)	↑ (n = 31)	↑ (n = 3)
L&M: MWM	↓ (n = 172)	↓ (n = 51)	– (n = 5)
L&M: Contextual fear conditioning test	↓ (n = 45)	↓ (n = 13)	↓ (n = 5)
L&M: Cued fear conditioning test	↓ (n = 40)	↓ (n = 9)	– (n = 3)
A: Elevated plus maze	↓ (n = 29)	*↓* (n = 17)	– (n = 2)
A: Open field test	– (n = 52)	– (n = 23)	– (n = 4)

ND, neurodegeneration related outcome; L&M, learning and memory related outcome; A, Anxiety related outcome. N represents the number of comparisons in the meta-analysis. ↑ = increased, ↓ = decreased, –= no effect.

Sevoflurane, isoflurane and desflurane all increase neuronal cell death. Behavioural changes were mainly observed in isoflurane and sevoflurane exposed animals. Learning and memory seems to be affected most often. Anxiety related outcomes do not seem to be influenced very much.

Table 2 summarizes the results from the subgroup analyses regarding duration of exposure and frequency of exposure. This table shows that the long term effects of sevoflurane and isoflurane on neurodegeneration could not be analysed due to too few studies in the long term subgroup. In other words; the neurodegenerative outcomes are in the majority of studies investigated relatively short term after exposure.

**Table 2:** Summary of the results from the subgroup analyses regarding timing of outcome assessment (short versus long term after exposure) and frequency of exposure (single versus multiple).

	Sevoflurane			Isoflurane			Sevoflurane			Isoflurane		
	Short	Long	∆	Short	Long	∆	Single	Mult	∆	Single	Mult	∆
ND: Caspase-3	↑		n.a	↑		n.a	↑	↑	NS	↑	↑	NS
ND: TUNEL	↑		n.a	↑		n.a	↑	↑	NS	↑	↑	n.a
L&M: MWM	↓	↓	NS	↓	↓	NS	↓	↓↓	S	↓↓	↓	S
L&M: Contextual fear conditioning test	↓	↓	NS		↓	n.a	↓	↓	NS			n.a
L&M: Cued fear conditioning test		↓	n.a			n.a	↓	↓	NS			n.a
A: Elevated plus maze	**–**	↓	NS			n.a	↓	↓	NS	**–**		n.a
A: Open field test	**–**	**–**	NS	**–**	**–**	NS	**–**	**–**	NS	**–**	**–**	NS

The presence of either an arrow or a dash indicates the direction of the effects within a subgroup. ↑ = increased, ↓ = decreased, –= no effect, ↓↓ = decreased more compared to the other group.

In case a cell is empty this indicates too few studies within a subgroup to conduct reliable analyses.

n.a. indicates that it was not possible to assess statistical differences between subgroups because of absence of at least 2 subgroups that were large enough.

Further, this table shows that sevoflurane seems to cause some long lasting effects on behaviour. Although a comparison between short and long term effects could not be executed, the subgroup containing the studies investigating relatively long term effects of sevoflurane was large enough, and revealed impaired learning and memory and increased anxiety. For isoflurane this was less pronounced and only observed in the Morris water maze test.

Finally this table shows that, except for the Morris water maze, we could not formally assess whether or not there exists a difference between single or multiple exposure to isoflurane. However, for most outcomes the subgroup of single exposure was large enough and reveals that single exposure to isoflurane increases neurodegeneration and diminishes learning and memory capabilities.

For sevoflurane exposure we could assess the difference between single versus multiple exposures in 6 out of the 7 outcomes. In general this difference was not significant. However, for all neurodegenerative and learning and memory outcomes single exposure increased neurodegeneration and impaired learning and memory.

### Sensitivity analyses

To test the robustness of the results for all halogenated ethers sensitivity analyses were observed. Removal of studies using medians instead of means did not alter the overall conclusion for either sevoflurane, isoflurane or desflurane. Also when we changed the cut-off values of the age categories, varied the categories for the timing of outcome assessment or removed extreme outliers (Hedges g larger than 20) our results appeared robust.

### Publication bias

The presence of possible publication bias was assessed for outcomes that contained at least 20 individual studies. As a consequence we did not assess the risk of publication bias for studies investigating the effect of desflurane.

Although visual inspection of the Funnel plots indicated some asymmetry, Duval and Tweedie’s trim and fill analysis and the Egger’s regression test did not show evidence for publication bias (supplemental file 10).

## Discussion

Although, the FDA concluded that the clinical significance of the nonclinical (animal) findings was not known (drug safety communication www.fda.gov/drugs/drugsafety/ucm532356.htm) at the time, the FDA issued a warning in 2016 regarding lengthy or repeated use of anesthetics and sedation drugs on brain development in children less than 3 years of age. To date the actual risk in human patients is still partly unclear. Some clinical studies showed that single exposure to sevoflurane and or isoflurane at an early age does not cause adverse neurodevelopmental effects^6–8^, but there are population based studies published that suggest the opposite^9^. Nevertheless, the clinical effect of repeated exposures and prolonged exposures to halogenated ethers remain completely unclear.

A systematic summary of all preclinical animal studies may provide insight. So far the current preclinical evidence base was poorly analyzed, and the animal studies the FDA based their warning on were generally not about the effects of halogenated ethers but more on ketamine and propofol.

We therefore evaluated in this study all preclinical evidence concerning isoflurane, sevoflurane, desflurane and enflurane exposure in young experimental animals on neurodegeneration and behaviour. We conducted subgroup analyses to investigate the effect of repeated versus single exposure and short versus prolonged exposure.

Our review showed exposure to sevoflurane, isoflurane and desflurane significantly increases neurodegeneration. Further, sevoflurane and isoflurane also cause learning and memory impairment, and increase anxiety. Desflurane showed little effect on learning and memory, and no effect on anxiety.

Subgroup analyses revealed that already shortly after exposure to isoflurane and sevoflurane neuronal cell death is present. Due to the limited number of studies available, and the majority of studies investigating short term effects, it is unclear if this damage is permanent and to what extend the brain will recover. However, we also show behavioural changes due to sevoflurane and isoflurane exposure, and the behavioural changes are measured somewhat later in time indicating some longer lasting and possibly permanent damage.

Further, this review showed that single exposure to either sevoflurane or isoflurane increased neurodegeneration and impaired learning and memory. This was most obvious for sevoflurane since all three learning and memory outcomes showed this. For isoflurane the single exposure subgroup was only large enough to draw reliable subgroup analyses for the Morris water Maze. More research into the effects of single exposure of isoflurane on (long lasting) behavioural changes is needed.

Nevertheless, this finding is partly in contrast with the warning issued by the FDA. The FDA stated that animal studies showed that single exposure to general anaesthetics are unlikely to have negative effects, whereas we observe both neurodegeneration and behavioural changes after single exposure. This could be explained by the fact that the FDA included only 19 animal studies in their reasoning, and we analysed a much larger evidence base (e.g. 324 studies). In addition, the 19 studies analysed by the FDA included many more anesthetic domains, they for example also analysed propfofol, ketamine and midazolam, which may have caused too much heterogeneity in the results, fading out the actual effects of halogenated ethers.

Taking into account the neurological damage shortly after exposure, and the longer lasting behavioural changes the use of sevoflurane and isoflurane should be restrained as much as possible in this young vulnerable group, until more research on the long term effect have been conducted.

For enflurane there were not enough studies to conduct meta analyses at all, and for desflurane due to the same reason no subgroup analyses could be conducted.

Obviously, more research into the effects of enflurane and desflurane is theoretically needed to explore whether the effects are similar to sevoflurane and isoflurane. It should be realized though, that these two halogenated ethers are currently of limited used in clinical practice, and even if found less harmful they would not become a preferred induction anesthetic agent in young children. Enflurane is very pungent and as a consequence not suitable for gas induction in young children. Desflurane requires a special electricity powered vaporizer and its price may render it unaffordable for many institutions. If all halogenated ethers prove, in both preclinical and clinical research, to be harmful for neuro development in children, possibly a shift should be made in the future towards intravenously induction of general anesthesia whenever possible.

### Strengths and limitations

This review screened over 30,000 references, and included 324 of them in this review. This very large evidence base, especially for isoflurane and sevoflurane, resulted in high precision and consequently improved the certainty in the body of evidence. In addition, our last update in December 2022, resulted in 82 additional papers, but the conclusions in this paper remained the same, underlining the robustness of the provided evidence.

Nevertheless, this review has also some important limitations. First of all we did not assess all available outcome measures related to neurodegeneration and behaviour. The variation in outcome measures was large and we focused on the most frequent used tests related to neurodegeneration and behaviour. In our view was pooling of all measures assessing various aspects related to neurodegeneration too heterogenous. We therefore focused on the 2 most frequently used (Tunel and Caspase) measures. Assessing all measures for neurodegeneration and techniques to assess behavioural changes individually may shine a different light on the results, and could be a topic for future research, although it is not expected that other neurodegenerative outcomes shine another light or are measured later in time compared to the Tunel and caspase assay.

Secondly, all but one of the studies that were included in this review used male animals. Females are greatly underrepresented. Although this is far from unique across research areas, this is problematic and reduces the construct validity and external validity. Future research should include both sexes.

Thirdly, our risk of bias analysis revealed that essential details regarding the design and conduct of the included experiments are poorly reported. As a consequence, the risk of bias could not be estimated in the majority of studies. Although this is no exception in this field, it is worrying as lack of reporting important methodological details will to some extent indicate neglected use of these methods to reduce bias causing skewed results^56^ and this may seriously hampers drawing reliable conclusions from the included animal studies.

Fourthly, analyses of the between study heterogeneity levels revealed moderate to severe levels of heterogeneity. Heterogeneity in animal research can be expected, as a result from the often-exploratory approach. In other words, part of the heterogeneity is intentionally induced^57^.

To account for anticipated heterogeneity, we used a random effects model, conducted sensitivity analyses and explored the suggested causes for between study heterogeneity by means of subgroup analyses. Exploring this heterogeneity is one of the added values of meta-analyses of animal studies and might help to inform the design of future animal studies and subsequent clinical trials.

Finally, this systematic review suffers from some indirectness. The majority of animal models studies assessed the effects on neurodegeneration and behaviour relatively short after exposure, whereas we are mainly interested in (long) lasting effects which remain relatively unsure. In addition, only a very limited number of studies investigated the effect of halogenated ethers in female animals.

## Conclusions and future recommendations

In summary, we show strong evidence that exposure to halogenated ethers causes neurodegeneration and behavioural changes in the developing animal brain. These effects are most pronounced for sevoflurane and isoflurane and already present after single exposure. To date there are not sufficient studies to estimate the presence of long term neurodegenerative effects. Nevertheless, we provide evidence in this review of behavioral changes that occur later in life due to exposure to halogenated ethers, suggesting some permanent neurodegenerative changes.

Altogether, in contrast to the warning issued by the FDA we show that already single exposure to isoflurane and sevoflurane negatively affects brain development in experimental animals. Based on the results of this review, the clinical use of sevoflurane and isoflurane should be restrained as much as possible in this young vulnerable group, until more research on the long term permanent effects have been conducted.

In order to further improve translation to the clinical situation it is also recommended to conduct studies with female animals and other species than rats as mice, because similar directions of effects in multiple species increases the chances of comparable results in humans^57^.

## Supplementary Information

Supplementary Information 1.

Supplementary Information 2.

Supplementary Information 3.

Supplementary Information 4.

Supplementary Information 5.

Supplementary Information 6.

Supplementary Information 7.

Supplementary Information 8.

Supplementary Information 9.

Supplementary Information 10.

## Data Availability

The majority of datasets generated during and/or analyzed during the conduct of this SR are available in the Supplementary Information files (file 4–9). The outcome data extracted from all original publications used in the meta analyses are available from the corresponding author on reasonable request.
