# Supplementary material for: Exposure to halogenated ethers causes neurodegeneration and behavioural changes in young healthy experimental animals: a systematic review and meta analyses
Source: Sci Rep. 2023 May 18;13:8063. doi: 10.1038/s41598-023-35052-4 (PMC10195874; doi:10.1038/s41598-023-35052-4)
Supplement: Supplementary file 1 — Supplementary Information 1. [file 41598_2023_35052_MOESM1_ESM.docx]

Supplemental file 1: Search strategy

**PubMed**- Age component
child [MeSh] OR Infant [MeSh] OR child* [tiab] OR newborn* [tiab] OR infant* [tiab] OR neonat* [tiab] OR prematur* [tiab] OR pre-matur* [tiab] OR immatur* [tiab] or juvenile* [tiab] OR Developmental [tiab] OR Baby [tiab] OR Babies [tiab] OR Young [tiab] OR perinat* [tiab] OR preterm* [tiab] OR pre-term* [tiab] OR prematur* [tiab] OR pre-matur* [tiab]

- General anesthetic component
((anesthesia, general [MeSh] OR anesthesia, intravenous [MeSh] OR anesthesiology [MeSh] OR anesthetics, general [MeSh] OR anesthetics, combined [MeSh] OR Anesthetics [Pharmacological Action] OR narcosis [tiab] OR anesthes* [tiab] OR anaesthes* [tiab] OR anesthet* [tiab] OR anaesthet* [tiab] OR neuroanesthes* [tiab] or neuroanaesthes* [tiab] OR neuroanesth* [tiab] OR neuroanaesth* [tiab]) OR (Desflurane [MeSh] OR Desflurane [tiab] OR Suprane [tiab] OR Isoflurane [MeSh] OR Isoflurane [tiab] OR Sevoflurane [MeSh] OR Sevoflurane [tiab] OR fluoromethyl [tiab] OR Hexafluoroisopropyl [tiab] OR Sevorane [tiab] OR Ultane [tiab] OR Nitrous oxide [MeSh] OR laughing gas [tiab] OR nitrogen protoxide [tiab] OR Enflurane [MeSh] OR enflurane [tiab] OR Etran [tiab] OR Ethrane [tiab] OR Chloroform [tiab] OR Ether [MeSh] OR ether [tiab] ethyl [tiab] OR Halothane [MeSh] OR Ftorotan [tiab] OR Narcotan [tiab] OR Fluothane [tiab] OR Methoxyflurane [MeSh] OR methoxyflurane [tiab] OR Penthrane [tiab] OR Pentrane [tiab] OR Anecotan [tiab] OR Trichloroethylene [tiab] OR Xenon [MeSh] OR Dexmedetomidine [MeSh] OR Dexmedetomidine [tiab] OR Precedex [tiab] OR Propofol [MeSH] OR Propofol [tiab] OR Diprivan [tiab] OR Disoprivan [tiab] OR Fresofol [tiab] OR Ivofol [tiab] OR Recofol [tiab] OR Aquafol [tiab] OR Thiopentone [MeSh] OR Thiopentone [tiab] OR Thiopental [MeSh] OR Thiopental [tiab] OR Thiomebumal [tiab] OR Penthiobarbital [tiab] OR Nesdonal [tiab] OR Pentothal [tiab] OR Sodipental [tiab] OR Thiopental [tiab] OR Nycomed [tiab] OR Trapanal [tiab] OR Bomathal [tiab] OR Thionembutal [tiab] OR Ketamine [MeSH] OR Ketamine [tiab] OR Ketalar [tiab] OR Ketaset [tiab] OR Ketanest [tiab] OR Calipsol [tiab] OR Kalipsol [tiab] OR Calypsol [tiab] OR Midazolam [MeSH] OR Midazolam [tiab] OR Methoexitone [tiab] OR methopenthal [tiab] OR Clonidine [MeSh] OR clonidine [tiab] OR Klofenil [tiab] OR Clofenil [tiab] OR Chlophazolin [tiab] OR Gemiton [tiab] OR Hemiton [tiab] OR Isoglaucon [tiab] OR Klofelin [tiab] OR Clofelin [tiab] OR Clopheline [tiab] OR Catapres [tiab] OR Catapresan [tiab] OR Catapressan [tiab] OR Dixarit [tiab] OR Trichloroethylene [MeSH] OR Trichloroethylene [tiab] OR Trichloroethene [tiab] OR Ethinyl Trichloride [tiab] OR Trielina [tiab] OR Trilene [tiab] OR Diazepam [MeSH] OR Diazepam [tiab] OR Diazemuls [tiab] OR Faustan [tiab] OR Valium [tiab] OR Seduxen [tiab] OR Sibazon [tiab] OR Stesolid [tiab] OR Apaurin [tiab] OR Relanium [tiab] OR Etomidate [MeSh] OR Ethomidate [tiab] OR Radenarkon [tiab] OR Hypnomidate [tiab] OR Methohexital [MeSh] OR methohexital [tiab] OR Methohexitone [tiab] OR Brevimytal [tiab] OR Natrium [tiab] OR Brevital [tiab] OR Brietal [tiab])

- Experimental animal component

Filter for retrieving all experimental animal studies in Pubmed (1)

**EMBASE**- Age component

child/ or infant/ or juvenile animal/ or exp neonatology/ or newborn/ or prematurity/ or baby/ or (child* or newborn* or infant* or neonat* or postmature* or prematur* or pre-matur* or juvenile* or Developmental or Baby or Babies or Young or perinat* or preterm* or pre-term* or prematur* or pre-matur*). ti,ab

- General anesthetic component
anesthetic agent/ or exp rectal anesthesia/ or exp intravenous anesthesia/ or anesthesiology/ or anesthetic agent/ or exp narcotic agent/ or (narcosis or anesthes* or anaesthes* or anesthet* or anaesthet* or neuroanesthes* or neuroanaesthes* or neuroanesth* or neuroanaesth*).ti,ab. or exp Desflurane/ or exp Sevoflurane/ or exp Nitrous oxide/ or exp Enflurane/ or exp Ether/ or exp Halothane/ or exp Methoxyflurane/ or exp Dexmedetomidine/ or exp Propofol/ or exp Etomidate/ or exp Methohexital/ or exp Thiopental/ or exp Esketamine/ or exp Morphine/ or exp Fentanyl/ or exp Oxycodone/ or exp Isoflurane/ or exp Midazolam/ or exp Diazepam/ or exp ketamine/ or exp remifentanil/ or (Desflurane or Suprane or Isoflurane or Sevoflurane or Sevorane or Ultane or laughing gas or nitrogen protoxide or enflurane or Etran or Ethrane or Chloroform or Ftorotan or Narcotan or Fluothane or methoxyflurane or Penthrane or Pentrane or Anecotan or Trichloroethylene or Dexmedetomidine or Precedex or Propofol or Disoprivan or Fresofol or Ivofol or Recofol or Aquafol or Ethomidate or Radenarkon or Hypnomidate or methohexital or Methohexitone or Brevimytal Natrium or Brevital or Brietal or Thiopentone or Thiopental or Thiomebumal or Nesdonal or Pentothal or Sodipental or Trapanal or Bomathal or Thionembutal or Esketamine or L-Ketamine or S-Ketamine or Morphine or Morphia or MS Contin or Oramorph SR or Duramorph or fentanyl or Phentanyl or Fentanest or Fentanyl Citrate or Sublimaze or Transmucosal Oral Fentanyl Citrate or Duragesic or Durogesic or Fentora or Sufentanil or Sulfentanyl or Sufenta or Alfentanil or Alfentanyl or Alfenta or Limifen or Rapifen or Fanaxal or Oxycodone or Dihydrone or Oxycone or Oxycodeinon or Eucodal or Theocodin or Oxycontin or Pancodine or Dinarkon or Oxiconum or Midazolam or Sulorane or Sevocalm or sevoflo or sevofrane or sevohale or sevotec or sojourn or Dinitrogen monoxide or dinitrogen oxide or factitious air or hyponitrous acid anhydride or Alyrane or compound 47 or efrane or enfran or gerolan or Anesthane or Fluorothan* or fluorhane or florotan or halan or halothane or ineltano or phthorothane or trothane or Anecotan or methoxy flurane or methoxyfluorane or metofan* or penthrox or Cepedex or dexamedetomidine or dexdomitor or dexdor or dexmedetomidine hydrochloride or primadex or sedadex or sileo or Anepol or cryotol or diisoprofol or diprivan or diprofol or disoprofol or gobbifol or pofol or propocam or rapinovet or safol or Amidate or radenarcon or Anesthal or farmotal or hypnostan or intraval or leopental or pentotal or penthiobarbital or pharmothal or ravonal or thionyl or thiotal or tio pental or tranpanal or S ketamin or vesierra or Anpec or duromorph or epimorph or miro or morfin* or morphi* or opso or skenan or epufen or fentalis or fentamyl or fentanil or fentanyl or fetanex or fetnanyl or leptanal or mezolar matrix or pecfent or rapiyl or recuvyra or subsys or tanyl or transfenta or Aerane or aerrane or forane or forene or forthane or isoflurano or isorane or sofloran or Dalam or buccolam or doricum or dormonid or fortanest or fulsed or hypnoval or hypnovel or hypnoyvel or ipnovel or midacum or midazo or midazol or midolam or miloz or versed or Imalgene or kalipsol or katamine or keta-hameln or ketaject or ketalar or ketalin or ketanest or ketased or ketaset or ketaved or ketavet or ketamin or ketoject or ketolar or narkamon or narketan or tekam or velonarcon or vetalar or etere etilico or ethoxy ethane or ethoxyethane or pronarco or bionine or bionone or bolodorm or broncodal or bucodal or cafacodal or cardanon or codenon or dihydrohydroxycodeinone or endone or eubine or eucodal or eucodale or eucodalum or eudin or eukdin or eukodal or eumorphal or eurodamine or eutagen or hydrocodal or hydroxycodeinoma or ludonal or medicdal or narcobasine or nargenol or narodal or nucodan or opton or ossicodone or oxanest or oxaydo or oxecta or oxicone or oxicotin or oxikon or oxycod or oxycodil or oxycone or oxycotin or oxydose or oxyfast or oxygesic or oxyir or oxykon or oxynorm or pancodine or pavinal or percolone or pronarcin or remoxy or roxicodone or roxycodone or sinthiodal or stupenal or supeudol or tebodal or tekodin or thecodin or xtampa or alboral or aliseum or alupram or amiprol or ansiolin or antenex or anxionil or apaurin or apaurine or apo-diazepam or armonil or azpozepam or arzepam or assival or atensine or audium or azedipamin or benzopin or betapam or bialzepam or bialzepan or calmpose or caudel or cercin or cercine or cersine or chlorodiazepam or compaz or desconet or diaceplex or dialag or dialar or diano or diapam or diapanil or diapax or diapin or diapine or diapo or diaquel or diastat or diazelium or diazem or diazemuls or diazepan or diazepin or diazidem or dipaz or dipezona or dizac or doval or drenian or ducene or dupin or duxen or eridan or eurosan or evacalm or fanstan or gewalcalm or gubex or kratium or lamra or lembrol or lipodiazepam or lorinon or lovium or melode or mentalium or methyldiazepinon* or morosan or neocalme or neurolytril or nivalenm noan or novazam or ortopsique or paceum or pacitran or paxum or plidan or propam or psychopax or radizepam or relanium or reliver or reposepan or saromet or sedapam or seduxen or serendil or setonil or simasedan or sipam or sonacon or stesolid or stesolin or tanquo tablinen or tensium or tranimul or tranquirit or tranquo puren or trazepam or umbrium or valaxona or valiquid or valium or valpam or valrelease or vanconin or vatran or vazen or vival or vivol or zetran or remifentanyl or ultiva or brevimytal or brevital or brieta or brietal or methohexal or methohexithone or methohexitol or methohexitone or methohexobarbital).ti,ab.

- Experimental animal component

Filter for retrieving all experimental animal studies in Embase (2)

References:

1. Hooijmans CR, Tillema A, Leenaars M, Ritskes-Hoitinga M. Enhancing search efficiency by means of a search filter for finding all studies on animal experimentation in PubMed. Laboratory animals. 2010;44(3):170-5.

2. de Vries RB, Hooijmans CR, Tillema A, Leenaars M, Ritskes-Hoitinga M. Updated version of the Embase search filter for animal studies. Laboratory animals. 2014;48(1):88.
