# Supplementary material for: Exposure to halogenated ethers causes neurodegeneration and behavioural changes in young healthy experimental animals: a systematic review and meta analyses
Source: Sci Rep. 2023 May 18;13:8063. doi: 10.1038/s41598-023-35052-4 (PMC10195874; doi:10.1038/s41598-023-35052-4)
Supplement: Supplementary file 2 — Supplementary Information 2. [file 41598_2023_35052_MOESM2_ESM.docx]

**Supplemental file 2**: Outcome data extraction of the measures used in behavioural tests

| Type of test | What does it measure | First preference for | Second preference for |
| --- | --- | --- | --- |
| Elevated plus maze | Anxiety | Time spent in open arm | Times open arm entered |
| Open field test | Anxiety related behaviour | Distance travelled | Time spent in centre |
| Morris water maze | Spatial learning and memory | Time spent in target quadrant | Platform crossings |
| Contextual fear conditioning | Hippocampal dependent learning and memory | Freezing response | - |
| Cued fear conditioning | Hippocampal independent learning and memory | Freezing response | - |
