# Supplementary material for: Exposure to halogenated ethers causes neurodegeneration and behavioural changes in young healthy experimental animals: a systematic review and meta analyses
Source: Sci Rep. 2023 May 18;13:8063. doi: 10.1038/s41598-023-35052-4 (PMC10195874; doi:10.1038/s41598-023-35052-4)
Supplement: Supplementary file 3 — Supplementary Information 3. [file 41598_2023_35052_MOESM3_ESM.docx]

**Supplemental file 3: Reference list of the 324 included studies**

[1-242] [243-324]

1. Almenrader, N., et al., *Effects of sevoflurane and clonidine on acid base status and long-term emotional and cognitive outcomes in spontaneously breathing rat pups.* PLoS ONE, 2017(e0173969).

2. Alvarado, M.C., K.L. Murphy, and M.G. Baxter, *Visual recognition memory is impaired in rhesus monkeys repeatedly exposed to sevoflurane in infancy.* Br J Anaesth, 2017. **119**(3): p. 517-523.

3. Bercker, S., et al., *Neurodegeneration in Newborn Rats Following Propofol and Sevoflurane Anesthesia.* Neurotoxicity Research, 2009(2): p. 140-147.

4. Bi, C., et al., *Sevoflurane induces neurotoxicity in the developing rat hippocampus by upregulating connexin 43 via the JNK/c-Jun/AP-1 pathway.* Biomed Pharmacother, 2018. **108**: p. 1469-1476.

5. Brambrink, A.M., et al., *Isoflurane-induced apoptosis of oligodendrocytes in the neonatal primate brain.* Annals of Neurology, 2012. **72**(4): p. 525-535.

6. Brambrink, A.M., et al., *Isoflurane-induced neuroapoptosis in the neonatal rhesus macaque brain.* Anesthesiology, 2010. **112**(4): p. 834-841.

7. Briner, A., et al., *Volatile anesthetics rapidly increase dendritic spine density in the rat medial prefrontal cortex during synaptogenesis.* Anesthesiology, 2010. **112**(3): p. 546-556.

8. Burks, S.M., et al., *Regions of the basal ganglia and primary olfactory system are most sensitive to neurodegeneration after extended sevoflurane anesthesia in the perinatal rat.* Neurotoxicology and Teratology, 2020. **80**.

9. Cao, W., et al., *Roles of aldosterone and oxytocin in abnormalities caused by sevoflurane anesthesia in neonatal rats.* Anesthesiology, 2012(4): p. 791-800.

10. Chen, C., et al., *Low-dose sevoflurane promotes hippocampal neurogenesis and facilitates the development of dentate gyrus-dependent learning in neonatal rats.* ASN Neuro, 2015. **7**(2).

11. Chen, X., et al., *Neonatal Exposure to Low-Dose (1.2%) Sevoflurane Increases Rats&amp;#039; Hippocampal Neurogenesis and Synaptic Plasticity in Later Life.* Neurotoxicity Research, 2018(2): p. 188-197.

12. Chen, Y., et al., *PDE-7 Inhibitor BRL-50481 Reduces Neurodegeneration and Long-Term Memory Deficits in Mice following Sevoflurane Exposure.* ACS Chemical Neuroscience, 2020. **11**(9): p. 1353-1358.

13. Chinn, G.A., et al., *Voluntary Exercise Rescues the Spatial Memory Deficit Associated With Early Life Isoflurane Exposure in Male Rats.* Anesth Analg, 2019. **129**(5): p. 1365-1373.

14. Chung, W., et al., *Sevoflurane exposure during the neonatal period induces long-term memory impairment but not autism-like behaviors.* Paediatric Anaesthesia, 2015(10): p. 1033-1045.

15. Coleman, K., et al., *Isoflurane Anesthesia Has Long-term Consequences on Motor and Behavioral Development in Infant Rhesus Macaques.* Anesthesiology, 2017(1): p. 74-84.

16. Dai, C.L., et al., *Neonatal Exposure to Anesthesia Leads to Cognitive Deficits in Old Age: Prevention with Intranasal Administration of Insulin in Mice.* Neurotoxicity Research, 2020. **38**(2): p. 299-311.

17. Demirgan, S., et al., *Isoflurane exposure in infant rats acutely increases aquaporin 4 and does not cause neurocognitive impairment.* Bosn J Basic Med Sci, 2019. **19**(3): p. 257-264.

18. Deng, C.Q., et al., *Effects of neonatal isoflurane exposures at different interval time on cognitive behavior in adult rats. [Chinese].* Medical Journal of Chinese People's Liberation Army, 2018. **43**(6): p. 465-471.

19. Deng, M., et al., *Brain regional vulnerability to anaesthesia-induced neuroapoptosis shifts with age at exposure and extends into adulthood for some regions.* Br J Anaesth, 2014. **113**(3): p. 443-51.

20. Dong, Y., et al., *Sevoflurane leads to learning and memory dysfunction via breaking the balance of tPA/PAI-1.* Neurochemistry International, 2020. **139**.

21. Dong, Y., et al., *Dexmedetomidine Attenuates Neurotoxicity in Developing Rats Induced by Sevoflurane through Upregulating BDNF-TrkB-CREB and Downregulating ProBDNF-P75NRT-RhoA Signaling Pathway.* Mediators of Inflammation, 2020. **2020**.

22. Drobish, J.K., et al., *Volatile anesthetics transiently disrupt neuronal development in neonatal rats.* Toxicological Sciences, 2016. **154**(2): p. 309-319.

23. Dydyk, L. and M. Justyna, *[Effect of enflurane on the motor cortex synapses in rabbits during the developmental period. Electron-microscopic studies by the routine method and after impregnation with ZIO].* Neuropatol Pol, 1981. **19**(2): p. 175-87.

24. Edwards, D.A., et al., *Bumetanide alleviates epileptogenic and neurotoxic effects of sevoflurane in neonatal rat brain.* Anesthesiology, 2010(3): p. 567-575.

25. Fan, X.Y., G. Shi, and P. Zhao, *Neonatal Sevoflurane Exposure Impairs Learning and Memory by the Hypermethylation of Hippocampal Synaptic Genes.* Molecular Neurobiology., 2020.

26. Fang, H., et al., *Repeated inhalation of sevoflurane inhibits the information transmission of Purkinje cells and delays motor development via the GABAA receptor epsilon subunit in neonatal mice.* Mol Med Rep, 2018(1): p. 1083-1092.

27. Fang, X., et al., *Chikusetsu saponin IVa attenuates isoflurane-induced neurotoxicity and cognitive deficits via SIRT1/ERK1/2 in developmental rats.* American Journal of Translational Research, 2017(9): p. 4288-4299.

28. Fang, X., et al., *Overexpression cdc42 attenuates isoflurane-induced neurotoxicity in developmental brain of rats.* Biochemical and Biophysical Research Communications, 2017(3): p. 719-725.

29. Feng, X., et al., *Single sevoflurane exposure decreases neuronal nitric oxide synthase levels in the hippocampus of developing rats.* British Journal of Anaesthesia, 2012. **109**(2): p. 225-233.

30. Fujimoto, S., et al., *Influence of neonatal sevoflurane exposure on nerve development-related microRNAs and behavior of rats.* Biomedical Research (Japan), 2015(6): p. 347-355.

31. Gao, J., et al., *Mdivi-1 pretreatment mitigates isoflurane-induced cognitive deficits in developmental rats.* American Journal of Translational Research, 2018(2): p. 432-443.

32. Gao, X., et al., *Sevoflurane enhanced the clearance of Abeta1-40 in hippocampus under surgery via up-regulating AQP-4 expression in astrocyte.* Life Sciences, 2019: p. 143-151.

33. Gentry, K.R., et al., *Early developmental exposure to volatile anesthetics causes behavioral defects in Caenorhabditis elegans.* Anesthesia and Analgesia, 2013(1): p. 185-189.

34. Goyagi, T., *The additional oxygen as a carrier gas during long-duration sevoflurane exposure ameliorate the neuronal apoptosis and improve the long-term cognitive function in neonatal rats.* Brain Res, 2018. **1678**: p. 220-230.

35. Han, X., et al., *Dexmedetomidine attenuates isoflurane-induced hippocampal neuroapoptosis through activation of Akt/Bad signaling pathways in neonatal rats. [Chinese].* Chinese Pharmacological Bulletin, 2013. **29**(12): p. 1702-1706.

36. Han, X., et al., *Calpain and JNK pathways participate in isoflurane - induced nucleus translocation of apoptosis-inducing factor in the brain of neonatal rats.* Toxicol Lett, 2018. **285**: p. 60-73.

37. Han, X., X. Zhang, and Z. Xue, *Sevoflurane induces long-term memory impairment and increases MeCP2 phosphorylation in developing mice.* International Journal of Clinical and Experimental Medicine, 2017. **10**(2): p. 2897-2903.

38. Hankenson, F.C., G.C. Braden-Weiss, and J.A. Blendy, *Behavioral and activity assessment of laboratory mice (Mus musculus) after tail biopsy under isoflurane anesthesia.* Journal of the American Association for Laboratory Animal Science, 2011. **50**(5): p. 686-694.

39. Head, B.P., et al., *Iof p75 neurotrophin receptor attenuates isoflurane-mediated neuronal apoptosis in the neonatal central nervous system.* Anesthesiology, 2009. **110**(4): p. 813-825.

40. Hu, X., et al., *Hydrogen sulfide attenuates isoflurane-induced neuroapoptosis and cognitive impairment in the developing rat brain.* BMC Anesthesiol, 2017. **17**(1): p. 123.

41. Huang, B.Y., et al., *Cell cycle activation contributes to isoflurane-induced neurotoxicity in the developing brain and the protective effect of CR8.* CNS Neurosci Ther, 2019. **25**(5): p. 612-620.

42. Huang, H., et al., *The Effects of Hesperidin on Neuronal Apoptosis and Cognitive Impairment in the Sevoflurane Anesthetized Rat are Mediated Through the PI3/Akt/PTEN and Nuclear Factor-κB (NF-κB) Signaling Pathways.* Med Sci Monit, 2020. **26**: p. e920522.

43. Huang, H., et al., *Repeated 2% sevoflurane administration in 7 and 60-day-old rats : Neurotoxicity and neurocognitive dysfunction.* Anaesthesist, 2017(11): p. 850-857.

44. Huaqin, S.U.N., et al., *Key miRNAs associated with memory and learning disorder upon exposure to sevoflurane determined by RNA sequencing.* Molecular Medicine Reports, 2020. **22**(2): p. 1567-1575.

45. Istaphanous, G.K., et al., *Comparison of the neuroapoptotic properties of equipotent anesthetic concentrations of desflurane, isoflurane, or sevoflurane in neonatal mice.* Anesthesiology, 2011. **114**(3): p. 578-587.

46. Ji, M.H., et al., *Pre-administration of curcumin prevents neonatal sevoflurane exposure-induced neurobehavioral abnormalities in mice.* NeuroToxicology, 2015: p. 155-164.

47. Ji, M.H., et al., *Environmental Enrichment Ameliorates Neonatal Sevoflurane Exposure-Induced Cognitive and Synaptic Plasticity Impairments.* Journal of Molecular Neuroscience, 2015(3): p. 358-365.

48. Ji, M.H., et al., *Repeated Neonatal Sevoflurane Exposure-Induced Developmental Delays of Parvalbumin Interneurons and Cognitive Impairments Are Reversed by Environmental Enrichment.* Molecular Neurobiology, 2017(5): p. 3759-3770.

49. Jia, M., et al., *Role of histone acetylation in long-term neurobehavioral effects of neonatal Exposure to sevoflurane in rats.* Neurobiology of Disease, 2016: p. 209-220.

50. Jiang, H., et al., *Hypoxia inducible factor-1alpha is involved in the neurodegeneration induced by isoflurane in the brain of neonatal rats.* J Neurochem, 2012. **120**(3): p. 453-60.

51. Jiang, J., et al., *Effect of multiple neonatal sevoflurane exposures on hippocampal apolipoprotein E levels and learning and memory abilities.* Pediatr Neonatol, 2018. **59**(2): p. 154-160.

52. Jiang, T., et al., *Genistein attenuates isoflurane-induced neurotoxicity and improves impaired spatial learning and memory by regulating cAMP/CREB and BDNF-TrkB-PI3K/Akt signaling.* Korean Journal of Physiology and Pharmacology, 2017(6): p. 579-589.

53. Jin, H., et al., *Paeonol attenuates isoflurane anesthesia-induced hippocampal neurotoxicity via modulation of JNK/ERK/P38MAPK pathway and regulates histone acetylation in neonatal rat.* Journal of Maternal Fetal and Neonatal Medicine., 2018.

54. Jin, X., et al., *Anesthesia plus surgery in neonatal period impairs preference for social novelty in mice at the juvenile age.* Biochemical and Biophysical Research Communications, 2020. **530**(3): p. 603-608.

55. Johnson, S.A., C. Young, and J.W. Olney, *Isoflurane-induced neuroapoptosis in the developing brain of nonhypoglycemic mice.* Journal of Neurosurgical Anesthesiology, 2008. **20**(1): p. 21-28.

56. Johnson, S.C., et al., *Relevance of experimental paradigms of anesthesia induced neurotoxicity in the mouse.* PLoS One, 2019. **14**(3): p. e0213543.

57. Ju, L., et al., *Subsequent maternal separation exacerbates neurobehavioral abnormalities in rats neonatally exposed to sevoflurane anesthesia.* Neuroscience Letters, 2017: p. 137-142.

58. Ju, L.S., et al., *Hypermethylation of Hippocampal Synaptic Plasticity-Related genes is Involved in Neonatal Sevoflurane Exposure-Induced Cognitive Impairments in Rats.* Neurotoxicity Research, 2016(2): p. 243-255.

59. Ju, L.S., et al., *Role of epigenetic mechanisms in transmitting the effects of neonatal sevoflurane exposure to the next generation of male, but not female, rats.* Br J Anaesth, 2018. **121**(2): p. 406-416.

60. Kilicaslan, A., et al., *Single and repeated sevoflurane or desflurane exposure does not impair spatial memory performance of young adult mice.* Fundamental and Clinical Pharmacology, 2013(6): p. 641-649.

61. Kodama, M., et al., *Neonatal desflurane exposure induces more robust neuroapoptosis than do isoflurane and sevoflurane and impairs working memory.* Anesthesiology, 2011. **115**(5): p. 979-991.

62. Kulak, A., et al., *Neurochemical profile of the developing mouse cortex determined by in vivo 1H NMR spectroscopy at 14.1 T and the effect of recurrent anaesthesia.* J Neurochem, 2010. **115**(6): p. 1466-77.

63. Landin, J.D., et al., *General anesthetic exposure in adolescent rats causes persistent maladaptations in cognitive and affective behaviors and neuroplasticity.* Neuropharmacology, 2019: p. 153-163.

64. Lee, B.H., et al., *Effect of combining anesthetics in neonates on long-term cognitive function.* Int J Dev Neurosci, 2014. **37**: p. 87-93.

65. Lee, J.H., et al., *The effect of lidocaine on apoptotic neurodegeneration in the developing mouse brain.* Korean J Anesthesiol, 2014. **67**(5): p. 334-41.

66. Lee, J.R., et al., *Alternative technique or mitigating strategy for sevoflurane-induced neurodegeneration: a randomized controlled dose-escalation study of dexmedetomidine in neonatal rats.* Br J Anaesth, 2017. **119**(3): p. 492-505.

67. Lei, X., et al., *Perinatal Supplementation with Omega-3 Polyunsaturated Fatty Acids Improves Sevoflurane-Induced Neurodegeneration and Memory Impairment in Neonatal Rats.* PLoS ONE, 2013(e70645).

68. Li, G., et al., *Developmental neurotoxicity in the context of multiple sevoflurane exposures: Potential role of histone deacetylase 6.* Neurotoxicol Teratol, 2019. **74**: p. 106813.

69. Li, G., et al., *S6 inhibition contributes to isoflurane neurotoxicity in the developing brain.* Toxicology Letters, 2015(2): p. 102-113.

70. Li, H., et al., *Intranasal Administration of Insulin Reduces Chronic Behavioral Abnormality and Neuronal Apoptosis Induced by General Anesthesia in Neonatal Mice.* Front Neurosci, 2019. **13**: p. 706.

71. Li, W., et al., *Rutin attenuates isoflurane-induced neuroapoptosis via modulating JNK and p38 MAPK pathways in the hippocampi of neonatal rats.* Experimental and Therapeutic Medicine, 2017(5): p. 2056-2064.

72. Li, X., et al., *Upregulation of Cdh1 Attenuates Isoflurane-Induced Neuronal Apoptosis and Long-Term Cognitive Impairments in Developing Rats.* Front Cell Neurosci, 2017. **11**(368): p. 368.

73. Li, Y., et al., *Sevoflurane induces short-term changes in proteins in the cerebral cortices of developing rats.* Acta Anaesthesiologica Scandinavica, 2013(3): p. 380-390.

74. Li, Y., et al., *Dexmedetomidine reduces isoflurane-induced neuroapoptosis partly by preserving PI3K/Akt pathway in the hippocampus of neonatal rats.* PLoS One, 2014. **9**(4): p. e93639.

75. Li, Y., et al., *Sevoflurane-induced learning deficits and spine loss via nectin-1/corticotrophin-releasing hormone receptor type 1 signaling.* Brain Res, 2019. **1710**: p. 188-198.

76. Liang, G., et al., *Isoflurane causes greater neurodegeneration than an equivalent exposure of sevoflurane in the developing brain of neonatal mice.* Anesthesiology, 2010. **112**(6): p. 1325-1334.

77. Liang, L., et al., *Involvement of homodomain interacting protein kinase 2-c-Jun N-terminal kinase/c-Jun cascade in the long-term synaptic toxicity and cognition impairment induced by neonatal Sevoflurane exposure.* J Neurochem, 2019.

78. Liang, X., et al., *Effect of repeated neonatal sevoflurane exposure on the learning, memory and synaptic plasticity at juvenile and adult age.* American Journal of Translational Research, 2017. **9**(11): p. 4974-4983.

79. Liao, Z., et al., *Both JNK and P38 MAPK pathways participate in the protection by dexmedetomidine against isoflurane-induced neuroapoptosis in the hippocampus of neonatal rats.* Brain Res Bull, 2014. **107**: p. 69-78.

80. Lin, D., et al., *Early-life single-episode sevoflurane exposure impairs social behavior and cognition later in life.* Brain and Behavior, 2016(e00514).

81. Lin, X.F., et al., *SAHA attenuates sevoflurane-induced learning and memory impairments in fetal mice.* Genetics and Molecular Research, 2014(4): p. 10769-10778.

82. Ling, Y., et al., *Sevoflurane exposure in postnatal rats induced long-term cognitive impairment through upregulating caspase-3/cleaved-poly (ADP-ribose) polymerase pathway.* Experimental and Therapeutic Medicine, 2017(4): p. 3824-3830.

83. Liu, B., et al., *Altered Metabolomic Profiles May Be Associated with Sevoflurane-Induced Neurotoxicity in Neonatal Rats.* Neurochemical Research., 2015.

84. Liu, B., et al., *Sevoflurane-Induced Endoplasmic Reticulum Stress Contributes to Neuroapoptosis and BACE-1 Expression in the Developing Brain: The Role of eIF2alpha.* Neurotoxicity Research, 2017(2): p. 218-229.

85. Liu, C.L., et al., *Effects of isoflurane and sevoflurane on Akt/GSK3beta pathway in neonatal rats brain. [Chinese].* Chinese Pharmaceutical Journal, 2011. **46**(13): p. 1003-1007.

86. Liu, F., et al., *Inhalation Anesthesia-Induced Neuronal Damage and Gene Expression Changes in Developing Rat Brain.* Syst Pharmacol, 2013. **1**: p. 1-9.

87. Liu, F., et al., *Potential Adverse Effects of Prolonged Sevoflurane Exposure on Developing Monkey Brain: From Abnormal Lipid Metabolism to Neuronal Damage.* Toxicological Sciences, 2015(2): p. 562-572.

88. Liu, G., et al., *Heightened stress response and cognitive impairment after repeated neonatal sevoflurane exposures might be linked to excessive GABAAR-mediated depolarization.* J Anesth, 2016(5): p. 834-41.

89. Liu, J., et al., *Roscovitine, a CDK5 Inhibitor, Alleviates Sevoflurane-Induced Cognitive Dysfunction via Regulation Tau/GSK3beta and ERK/PPARgamma/CREB Signaling.* Cellular Physiology and Biochemistry, 2017(2): p. 423-435.

90. Liu, J., et al., *Neonatal Repeated Exposure to Isoflurane not Sevoflurane in Mice Reversibly Impaired Spatial Cognition at Juvenile-Age.* Neurochem Res, 2017. **42**(2): p. 595-605.

91. Liu, T.D., K. Yang, and F.K. Li, *Neuroprotective effects of pterostilbene against isoflurane-induced apoptosis through regulating the JNK and PI3K/Akt pathway in neonatal rats.* Bangladesh Journal of Pharmacology, 2015(1): p. 173-182.

92. Liu, Y., et al., *Influence of sevoflurane exposure on mitogen-activated protein kinases and Akt/GSK-3beta/CRMP-2 signaling pathways in the developing rat brain.* Experimental and Therapeutic Medicine, 2018(2): p. 2066-2073.

93. Loepke, A.W., et al., *The effects of neonatal isoflurane exposure in mice on brain cell viability, adult behavior, learning, and memory.* Anesthesia and Analgesia, 2009. **108**(1): p. 90-104.

94. Lu, H., et al., *Sevoflurane Acts on Ubiquitination-Proteasome Pathway to Reduce Postsynaptic Density 95 Protein Levels in Young Mice.* Anesthesiology, 2017. **127**(6): p. 961-975.

95. Lu, Y., et al., *Anesthetic sevoflurane causes neurotoxicity differently in neonatal naive and alzheimer disease transgenic mice.* Anesthesiology, 2010(6): p. 1404-1416.

96. Luo, R.Y., et al., *Early-Life Multiple Sevoflurane Exposures Alleviate Long-term Anxiety-Like Behaviors in Mice via the proBDNF/ERK Pathway.* Molecular Neurobiology., 2020.

97. Ma, D., et al., *Xenon mitigates isoflurane-induced neuronal apoptosis in the developing rodent brain.* Anesthesiology, 2007. **106**(4): p. 746-753.

98. Ma, J.F., et al., *Isoflurane and sevoflurane affects Wnt/beta-catenin signaling pathways in hippocampal formation of neonatal rats.* European review for medical and pharmacological sciences, 2017(8): p. 1980-1989.

99. Ma, R., et al., *alpha-Lipoic acid inhibits sevoflurane-induced neuronal apoptosis through PI3K/Akt signalling pathway.* Cell Biochemistry and Function, 2016(1): p. 42-47.

100. Makaryus, R., et al., *Brain maturation in neonatal rodents is impeded by sevoflurane anesthesia.* Anesthesiology, 2015. **123**(3): p. 557-68.

101. Makaryus, R., et al., *Noninvasive Tracking of Anesthesia Neurotoxicity in the Developing Rodent Brain.* Anesthesiology, 2018. **129**(1): p. 118-130.

102. Maloney, S.E., et al., *Repeated neonatal isoflurane exposures in the mouse induce apoptotic degenerative changes in the brain and relatively mild long-term behavioral deficits.* Sci Rep, 2019. **9**(1): p. 2779.

103. Man, Y.G., R.G. Zhou, and B. Zhao, *Efficacy of rutin in inhibiting neuronal apoptosis and cognitive disturbances in sevoflurane or propofol exposed neonatal mice.* Int J Clin Exp Med, 2015. **8**(8): p. 14397-409.

104. McAuliffe, J.J., B. Joseph, and C.V. Vorhees, *Isoflurane-delayed preconditioning reduces immediate mortality and improves striatal function in adult mice after neonatal hypoxia-ischemia.* Anesthesia and Analgesia, 2007. **104**(5): p. 1066-1077.

105. Medeiros, L.F., et al., *Lifetime behavioural changes after exposure to anaesthetics in infant rats.* Behavioural Brain Research, 2011. **218**(1): p. 51-56.

106. Miao, L.P., et al., *Effects of lithium chloride on isoflurane-induced neuroapoptosis in neonatal rat hippocampus. [Chinese].* Chinese Pharmaceutical Journal, 2016. **51**(22): p. 1923-1928.

107. Murphy, K.L. and M.G. Baxter, *Long-term effects of neonatal single or multiple isoflurane exposures on spatial memory in rats.* Front Neurol, 2013. **4**: p. 87.

108. Murphy, K.L., et al., *Exposure to sevoflurane anesthesia during development does not impair aspects of attention during adulthood in rats.* Neurotoxicol Teratol, 2017. **60**: p. 87-94.

109. Nakamura, E., et al., *Sevoflurane inhalation accelerates the long-term memory consolidation via small gtpase overexpression in the hippocampus of mice in adolescence.* PLoS ONE, 2016(e0163151).

110. Neira, A., et al., *Effects of multiple exposures to sevoflurane at sub-MAC doses on neuroapoptosis and cognitive function during the neonatal period. [Spanish].* Revista Colombiana de Anestesiologia, 2014(3): p. 154-165.

111. Noguchi, K.K., et al., *Lithium Protects Against Anaesthesia Neurotoxicity In The Infant Primate Brain.* Sci Rep, 2016. **6**: p. 22427.

112. O'Farrell, R.A., et al., *Neurotoxicity of Inhalation Anesthetics in the Neonatal Rat Brain: Effects on Behavior and Neurodegeneration in the Piriform Cortex.* Anesthesiol Res Pract, 2018. **2018**(6376090): p. 6376090.

113. Ozer, A.B., et al., *Effects of sevoflurane on apoptosis, BDNF and cognitive functions in neonatal rats.* Bratisl Lek Listy, 2017. **118**(2): p. 80-84.

114. Pan, B., et al., *The neuroprotective effects of remifentanil on isoflurane-induced apoptosis in the neonatal rat brain.* Am J Transl Res, 2017. **9**(10): p. 4521-4533.

115. Pang, X., et al., *Dexmedetomidine pretreatment attenuates isoflurane-induced neurotoxicity via inhibiting the TLR2/NF-kappaB signaling pathway in neonatal rats.* Exp Mol Pathol, 2020. **112**: p. 104328.

116. Pellegrini, L., et al., *Erythropoietin protects newborn rat against sevoflurane-induced neurotoxicity.* Paediatric Anaesthesia, 2014(7): p. 749-759.

117. Peng, J., et al., *Anesthetic preconditioning inhibits isoflurane-mediated apoptosis in the developing rat brain.* Anesth Analg, 2014. **119**(4): p. 939-46.

118. Perez-Zoghbi, J.F., et al., *Dexmedetomidine-mediated neuroprotection against sevoflurane-induced neurotoxicity extends to several brain regions in neonatal rats.* Br J Anaesth, 2017. **119**(3): p. 506-516.

119. Perez-Zoghbi, J.F., et al., *Neurotoxicity of sub-anesthetic doses of sevoflurane and dexmedetomidine co-administration in neonatal rats.* NeuroToxicology, 2020. **79**: p. 75-83.

120. Qin, J.H., et al., *Effect of sevoflurane and halothane anesthesia on cognitive function and immune function in young rats.* Saudi J Biol Sci, 2018. **25**(1): p. 47-51.

121. Qiu, L., et al., *Acute and Long-Term Effects of Brief Sevoflurane Anesthesia During the Early Postnatal Period in Rats.* Toxicological Sciences, 2016(1): p. 121-133.

122. Ramage, T.M., et al., *Distinct long-term neurocognitive outcomes after equipotent sevoflurane or isoflurane anaesthesia in immature rats.* Br J Anaesth, 2013. **110**: p. i39-46.

123. Raper, J., et al., *Multiple Anesthetic Exposure in Infant Monkeys Alters Emotional Reactivity to an Acute Stressor.* Anesthesiology, 2015. **123**(5): p. 1084-92.

124. Raper, J., et al., *Multiple sevoflurane exposures in infant monkeys do not impact the mother-infant bond.* Neurotoxicol Teratol, 2016. **54**: p. 46-51.

125. Raper, J., et al., *Persistent alteration in behavioural reactivity to a mild social stressor in rhesus monkeys repeatedly exposed to sevoflurane in infancy.* Br J Anaesth, 2018. **120**(4): p. 761-767.

126. Rosado-Mendez, I.M., et al., *Quantitative ultrasound and apoptotic death in the neonatal primate brain.* Neurobiol Dis, 2019. **127**: p. 554-562.

127. Rosenholm, M., et al., *Repeated brief isoflurane anesthesia during early postnatal development produces negligible changes on adult behavior in male mice.* PLoS One, 2017. **12**(4): p. e0175258.

128. Rothstein, S., T. Simkins, and J.L. Nunez, *Response to neonatal anesthesia: Effect of sex on anatomical and behavioral outcome.* Neuroscience, 2008. **152**(4): p. 959-969.

129. Sanders, R.D., et al., *Dexmedetomidine attenuates isoflurane-induced neurocognitive impairment in neonatal rats.* Anesthesiology, 2009. **110**(5): p. 1077-1085.

130. Satomoto, M., et al., *Neonatal exposure to sevoflurane induces abnormal social behaviors and deficits in fear conditioning in Mice.* Anesthesiology, 2009(3): p. 628-637.

131. Satomoto, M., et al., *Sugammadex-Enhanced Neuronal Apoptosis following Neonatal Sevoflurane Exposure in Mice.* Anesthesiology Research and Practice, 2016(9682703).

132. Satomoto, M., et al., *Neonatal Sevoflurane Exposure Induces Adulthood Fear-induced Learning Disability and Decreases Glutamatergic Neurons in the Basolateral Amygdala.* J Neurosurg Anesthesiol, 2018. **30**(1): p. 59-64.

133. Schaefer, M.L., et al., *Neonatal isoflurane anesthesia or disruption of postsynaptic density-95 protein interactions change dendritic spine densities and cognitive function in juvenile mice.* Anesthesiology, 2020: p. 812-823.

134. Schaefer, M.L., et al., *Nitric Oxide Donor Prevents Neonatal Isoflurane-induced Impairments in Synaptic Plasticity and Memory.* Anesthesiology, 2019. **130**(2): p. 247-262.

135. Sen, T. and N. Sen, *Isoflurane-induced inactivation of CREB through histone deacetylase 4 is responsible for cognitive impairment in developing brain.* Neurobiol Dis, 2016. **96**: p. 12-21.

136. Seubert, C.N., et al., *Developmental effects of neonatal isoflurane and sevoflurane exposure in rats.* Anesthesiology, 2013. **119**(2): p. 358-64.

137. Shen, F.Y., et al., *Role of T-type Calcium Channels in Generating Hyperexcitatory Behaviors during Emergence from Sevoflurane Anesthesia in Neonatal Rats.* Neurosci Bull, 2020.

138. Shen, F.Y., et al., *Cognitive impairment and endoplasmic reticulum stress induced by repeated short-term sevoflurane exposure in early life of rats.* Frontiers in Psychiatry, 2018(332).

139. Shen, X., et al., *Selective anesthesia-induced neuroinflammation in developing mouse brain and cognitive impairment.* Anesthesiology, 2013. **118**(3): p. 502-15.

140. Shen, X., et al., *Early life exposure to sevoflurane impairs adulthood spatial memory in the rat.* Neurotoxicology, 2013. **39**: p. 45-56.

141. Shi, Y., et al., *Hydrogen gas attenuates sevoflurane neurotoxicity through inhibiting nuclear factor kappa-light-chain-enhancer of activated B cells signaling and proinflammatory cytokine release in neonatal rats.* Neuroreport, 2017. **28**(17): p. 1170-1175.

142. Shih, J., et al., *Delayed environmental enrichment reverses sevoflurane-induced memory impairment in rats.* Anesthesiology, 2012(3): p. 586-602.

143. Shuai, Y.-F., et al., *Effects of vitamin C on inhalation anesthetic isoflurane-induced developmental, neuronal apoptosis in neonatal rats.* Bangladesh Journal of Pharmacology, 2014. **9**(4): p. 580-587.

144. Song, Q., et al., *Sevoflurane induces neurotoxicity in young mice through FAS/FASL signaling.* Genetics and Molecular Research, 2015(4): p. 18059-18068.

145. Song, S.Y., et al., *Cognitive impairment and transcriptomic profile in hippocampus of young mice after multiple neonatal exposures to sevoflurane.* Aging (Albany NY), 2019. **11**(19): p. 8386-8417.

146. Stratmann, G., et al., *Effect of general anesthesia in infancy on long-term recognition memory in humans and rats.* Neuropsychopharmacology, 2014. **39**(10): p. 2275-87.

147. Stratmann, G., et al., *Isoflurane differentially affects neurogenesis and long-term neurocognitive function in 60-day-old and 7-day-old rats.* Anesthesiology, 2009. **110**(4): p. 834-48.

148. Sun, G.Y., et al., *Sevoflurane induces temporary spatial working memory deficits and synaptic ultrastructure impairments in the hippocampus of neonatal rats.* Eur Rev Med Pharmacol Sci, 2019. **23**(6): p. 2620-2629.

149. Sun, Z., et al., *Inhibiting NADPH oxidase protects against long-term memory impairment induced by neonatal sevoflurane exposure in mice.* Br J Anaesth, 2016. **117**(1): p. 80-6.

150. Sun, Z., M. Satomoto, and K. Makita, *Therapeutic effects of intravenous administration of bone marrow stromal cells on sevoflurane-induced neuronal apoptosis and neuroinflammation in neonatal rats.* Korean Journal of Anesthesiology, 2015(4): p. 397-401.

151. Tagawa, T., et al., *Sevoflurane in combination with propofol, not thiopental, induces a more robust neuroapoptosis than sevoflurane alone in the neonatal mouse brain.* Journal of Anesthesia, 2014(6): p. 815-820.

152. Takaenoki, Y., et al., *Neonatal exposure to sevoflurane in mice causes deficits in maternal behavior later in adulthood.* Anesthesiology, 2014. **120**(2): p. 403-15.

153. Tan, L., et al., *Pharmacological inhibition of PTEN attenuates cognitive deficits caused by neonatal repeated exposures to isoflurane via inhibition of NR2B-mediated tau phosphorylation in rats.* Neuropharmacology, 2017. **114**: p. 135-145.

154. Tan, Y.T., et al., *Effects of resveratrol on the cognitive function of neonate rats anesthetized by sevoflurane exposure. [Chinese].* Medical Journal of Chinese People's Liberation Army, 2018. **43**(7): p. 600-606.

155. Tang, X., et al., *Role of alpha7nAChR-NMDAR in sevoflurane-induced memory deficits in the developing rat hippocampus.* PLoS One, 2018(2): p. e0192498.

156. Tang, X., et al., *NR2B receptor- and calpain-mediated KCC2 cleavage resulted in cognitive deficiency exposure to isoflurane.* Neurotoxicology, 2020. **76**: p. 75-83.

157. Tao, G., et al., *Docosahexaenoic Acid Rescues Synaptogenesis Impairment and Long-Term Memory Deficits Caused by Postnatal Multiple Sevoflurane Exposures.* BioMed Research International, 2016(4062579).

158. Tao, G., et al., *Isoflurane Is More Deleterious to Developing Brain Than Desflurane: The Role of the Akt/GSK3beta Signaling Pathway.* Biomed Res Int, 2016. **2016**(7919640): p. 7919640.

159. Walters, J.L., et al., *Acetyl-L-carnitine does not prevent neurodegeneration in a rodent model of prolonged neonatal anesthesia.* Neurotoxicology and Teratology, 2020. **80**.

160. Wang, C., et al., *Lipidomics reveals a systemic energy deficient state that precedes neurotoxicity in neonatal monkeys after sevoflurane exposure.* Anal Chim Acta, 2018. **1037**: p. 87-96.

161. Wang, D., et al., *Betulinic acid protects the neuronal damage in new born rats from isoflurane-induced apoptosis in the developing brain by blocking FASL-FAS signaling pathway.* Biomed Pharmacother, 2017. **95**: p. 1631-1635.

162. Wang, J., et al., *The Estradiol Synthesis Inhibitor Formestane Diminishes the Ability of Sevoflurane to Induce Neurodevelopmental Abnormalities in Male Rats.* Frontiers in Systems Neuroscience, 2020. **14**.

163. Wang, L., et al., *Carbon monoxide incompletely prevents isoflurane-induced defects in murine neurodevelopment.* Neurotoxicol Teratol, 2017. **61**: p. 92-103.

164. Wang, L.Y., Z.J. Tang, and Y.Z. Han, *Neuroprotective effects of caffeic acid phenethyl ester against sevoflurane-induced neuronal degeneration in the hippocampus of neonatal rats involve MAPK and PI3K/Akt signaling pathways.* Molecular Medicine Reports, 2016(4): p. 3403-3412.

165. Wang, S.Q., et al., *Neonatal sevoflurane anesthesia induces long-term memory impairment and decreases hippocampal PSD-95 expression without neuronal loss.* European Review for Medical and Pharmacological Sciences, 2013. **17**(7): p. 941-950.

166. Wang, W., et al., *Glycyrrhizin attenuates isoflurane-induced cognitive deficits in neonatal rats via its anti-inflammatory activity.* Neuroscience, 2016. **316**: p. 328-36.

167. Wang, W.Y., et al., *Location- and Subunit-Specific NMDA Receptors Determine the Developmental Sevoflurane Neurotoxicity Through ERK1/2 Signaling.* Molecular Neurobiology, 2016(1): p. 216-230.

168. Wang, W.Y., et al., *Inhibition of aberrant cyclin-dependent kinase 5 activity attenuates isoflurane neurotoxicity in the developing brain.* Neuropharmacology, 2014: p. 90-99.

169. Wang, W.Y., et al., *The effects of metabotropic glutamate receptor 7 allosteric agonist N,N'-dibenzhydrylethane-1,2-diamine dihydrochloride on developmental sevoflurane neurotoxicity: Role of extracellular signal-regulated kinase 1 and 2 mitogen-activated protein kinase signaling pathway.* Neuroscience, 2012. **205**: p. 167-177.

170. Wang, W.Y., et al., *Beta-arrestin1 and 2 differently modulate metabotropic glutamate receptor 7 signaling in rat developmental sevoflurane-induced neuronal apoptosis.* Neuroscience, 2016. **313**: p. 199-212.

171. Wang, W.Y., et al., *N-stearoyl-L-tyrosine ameliorates sevoflurane induced neuroapoptosis via MEK/ERK1/2 MAPK signaling pathway in the developing brain.* Neurosci Lett, 2013. **541**: p. 167-72.

172. Wang, X., et al., *Sevoflurane induces cognitive impairment in young mice via autophagy.* PLoS One, 2019. **14**(5): p. e0216372.

173. Wang, X., et al., *Neuroprotective effects of dexmedetomidine against isoflurane-induced neuronal injury via glutamate regulation in neonatal rats.* Drug Des Devel Ther, 2019. **13**: p. 153-160.

174. Wang, Y., et al., *Pre-administration of luteoline attenuates neonatal sevoflurane-induced neurotoxicity in mice.* Acta Histochem, 2019. **121**(4): p. 500-507.

175. Wei, K., et al., *Defining the Vulnerability Window of Anesthesia-Induced Neuroapoptosis in Developing Dentate Gyrus Granule Cells - A Transgenic Approach Utilizing POMC-EGFP Mice.* Neuroscience, 2019. **415**: p. 59-69.

176. Wirak, G.S., C.V. Gabel, and C.W. Connor, *Isoflurane Exposure in Juvenile Caenorhabditis elegans Causes Persistent Changes in Neuron Dynamics.* Anesthesiology, 2020. **133**(3): p. 569-582.

177. Wu, J., et al., *Iron overload contributes to general anaesthesia-induced neurotoxicity and cognitive deficits.* Journal of Neuroinflammation, 2020. **17**(1).

178. Wu, Z., et al., *Effects of early postnatal sevoflurane exposure on oligodendrocyte maturation and myelination in cerebral white matter of the rat.* Biomedicine and Pharmacotherapy, 2020. **131**.

179. Wu, Z., et al., *Sirtuin 2 Inhibition Attenuates Sevoflurane-Induced Learning and Memory Deficits in Developing Rats via Modulating Microglial Activation.* Cell Mol Neurobiol, 2020. **40**(3): p. 437-446.

180. Xia, Y., et al., *Tanshinone IIA Attenuates Sevoflurane Neurotoxicity in Neonatal Mice.* Anesth Analg, 2017. **124**(4): p. 1244-1252.

181. Xiao, H., et al., *Learning, memory and synaptic plasticity in hippocampus in rats exposed to sevoflurane.* Int J Dev Neurosci, 2016. **48**: p. 38-49.

182. Xie, L., et al., *Neonatal sevoflurane exposure induces impulsive behavioral deficit through disrupting excitatory neurons in the medial prefrontal cortex in mice.* Translational Psychiatry, 2020. **10**(1).

183. Xu, C., et al., *MicroRNA-96 is responsible for sevoflurane-induced cognitive dysfunction in neonatal rats via inhibiting IGF1R.* Brain Res Bull, 2019. **144**: p. 140-148.

184. Xu, C., et al., *Anesthesia with sevoflurane in neonatal rats: Developmental neuroendocrine abnormalities and alleviating effects of the corticosteroid and Cl(-) importer antagonists.* Psychoneuroendocrinology, 2015. **60**: p. 173-81.

185. Xu, G., et al., *Coenzyme Q10 reduces sevoflurane-induced cognitive deficiency in young mice.* Br J Anaesth, 2017. **119**(3): p. 481-491.

186. Xu, K.X., et al., *Neuroprotective properties of vitamin C on equipotent anesthetic concentrations of desflurane, isoflurane, or sevoflurane in high fat diet fed neonatal mice.* International Journal of Clinical and Experimental Medicine, 2015(7): p. 10444-10458.

187. Xu, L., et al., *Autophagy is involved in sevoflurane-induced developmental neurotoxicity in the developing rat brain.* Brain Res Bull, 2018. **140**: p. 226-232.

188. Xu, L., et al., *MicroRNA-325-3p prevents sevoflurane-induced learning and memory impairment by inhibiting Nupr1 and C/EBPbeta/IGFBP5 signaling in rats.* Aging (Albany NY), 2020. **12**(6): p. 5209-5220.

189. Xu, N., et al., *A Methyltransferase Inhibitor (Decitabine) Alleviates Intergenerational Effects of Paternal Neonatal Exposure to Anesthesia with Sevoflurane.* Anesthesia and Analgesia, 2020: p. 1291-1299.

190. Xu, X., et al., *The decrease of NMDAR subunit expression and NMDAR EPSC in hippocampus by neonatal exposure to desflurane in mice.* Behav Brain Res, 2017. **317**: p. 82-87.

191. Xue, H., et al., *Sevoflurane post-conditioning alleviates neonatal rat hypoxic-ischemic cerebral injury via Ezh2-regulated autophagy.* Drug Des Devel Ther, 2019. **13**: p. 1691-1706.

192. Yahalom, B., et al., *Spinal anesthesia in infant rats: development of a model and assessment of neurologic outcomes.* Anesthesiology, 2011. **114**(6): p. 1325-35.

193. Yang, B., et al., *Comparison of neurodegeneration and cognitive impairment in neonatal mice exposed to propofol or isoflurane.* PLoS One, 2014. **9**(6): p. e99171.

194. Yang, F., et al., *The Neuroprotective Effect of Hemin and the Related Mechanism in Sevoflurane Exposed Neonatal Rats.* Front Neurosci, 2019. **13**: p. 537.

195. Yang, F., et al., *Hemin treatment protects neonatal rats from sevoflurane-induced neurotoxicity via the phosphoinositide 3-kinase/Akt pathway.* Life Sci, 2020. **242**: p. 117151.

196. Yang, J., et al., *Acute injury to cerebral function produced by isoflurane anesthesia given at different time interval in neonatal rats. [Chinese].* Zhonghua Wei Zhong Bing Ji Jiu Yi Xue, 2016. **28**(3): p. 267-271.

197. Yang, L., et al., *Sevoflurane induces neuronal activation and behavioral hyperactivity in young mice.* Scientific reports, 2020. **10**(1): p. 11226.

198. Yang, W., et al., *microRNA-124 attenuates isoflurane-induced neurological deficits in neonatal rats via binding to EGR1.* J Cell Physiol, 2019. **234**(12): p. 23017-23032.

199. Yang, X., et al., *Downregulation of CDK5 Restores Sevoflurane-Induced Cognitive Dysfunction by Promoting SIRT1-Mediated Autophagy.* Cell Mol Neurobiol, 2020.

200. Yang, Z.J., et al., *Pre-treatment with a Xingnaojing preparation ameliorates sevoflurane-induced neuroapoptosis in the infant rat striatum.* Molecular Medicine Reports, 2015(3): p. 1615-1622.

201. Yi, X., Y. Cai, and W. Li, *Isoflurane Damages the Developing Brain of Mice and Induces Subsequent Learning and Memory Deficits through FASL-FAS Signaling.* Biomed Res Int, 2015. **2015**(315872): p. 315872.

202. Yonamine, R., et al., *Coadministration of hydrogen gas as part of the carrier gas mixture suppresses neuronal apoptosis and subsequent behavioral deficits caused by neonatal exposure to sevoflurane in mice.* Anesthesiology, 2013(1): p. 105-113.

203. Yong, J., et al., *Effects of compound 21, a nonpeptide angiotensin II type 2 receptor agonist, on general anesthesiainduced cerebral injury in neonatal rats.* Mol Med Rep, 2018. **18**(6): p. 5337-5344.

204. Yu, X., et al., *Effects of sevoflurane on learning, memory, and expression of pERK1/2 in hippocampus in neonatal rats.* Acta Anaesthesiologica Scandinavica, 2015(1): p. 78-84.

205. Yu, X., F. Zhang, and J. Shi, *Neonatal exposure to sevoflurane caused cognitive deficits by dysregulating SK2 channels and GluA2-lacking AMPA receptors in juvenile rat hippocampus.* Neuropharmacology, 2018. **141**: p. 66-75.

206. Yu, Y., et al., *Tau Contributes to Sevoflurane-induced Neurocognitive Impairment in Neonatal Mice.* Anesthesiology, 2020. **133**(3): p. 595-610.

207. Yu, Y., et al., *Sevoflurane induces cognitive impairments via the MiR-27b/LIMK1-signaling pathway in developing rats.* Inhalation Toxicology, 2016(14): p. 731-738.

208. Yu, Z.P., et al., *Neurotoxic effects of sevoflurane and isoflurane acute exposure on hippocampus of neonatal SD rats. [Chinese].* Chinese Journal of Pharmacology and Toxicology, 2011. **25**(1): p. 72-76.

209. Yu, Z.P., et al., *Effect of acute exposure to sevoflurane and isoflurane on learning, memory and brain derived neurotrophic factor expression in hippocampus of juvenile SD rats. [Chinese].* Chinese Journal of Pharmacology and Toxicology, 2013. **27**(2): p. 132-137.

210. Yuede, C.M., J.W. Olney, and C.E. Creeley, *Developmental neurotoxicity of alcohol and anesthetic drugs is augmented by co-exposure to caffeine.* Brain Sci, 2013. **3**(3): p. 1128-52.

211. Yufune, S., et al., *Suppression of ERK phosphorylation through oxidative stress is involved in the mechanism underlying sevoflurane-induced toxicity in the developing brain.* Scientific reports, 2016: p. 21859.

212. Zeng, M.T., et al., *Effects of dexmedetomidine on isoflurane-induced neuroapoptosis and expression of CRMP2 in neonatal rat hippocampus. [Chinese].* Chinese Pharmaceutical Journal, 2013. **48**(14): p. 1165-1169.

213. Zhang, B.J. and C.X. Yuan, *Effects of ADAM2 silencing on isoflurane-induced cognitive dysfunction via the P13K/Akt signaling pathway in immature rats.* Biomed Pharmacother, 2019. **109**: p. 217-225.

214. Zhang, J., et al., *Interaction of Tau, IL-6 and mitochondria on synapse and cognition following sevoflurane anesthesia in young mice.* Brain, Behavior, and Immunity - Health, 2020. **8**.

215. Zhang, J., et al., *Anesthetic Sevoflurane Reduces Levels of Hippocalcin and Postsynaptic Density Protein 95.* Molecular Neurobiology, 2015(3): p. 853-863.

216. Zhang, J.X., et al., *Neuroprotective effect of paeonol against isofluraneinduced neuroapoptosis and cognitive dysfunction.* Tropical Journal of Pharmaceutical Research, 2016(10): p. 2173-2182.

217. Zhang, L., et al., *Disrupted folate metabolism with anesthesia leads to myelination deficits mediated by epigenetic regulation of ERMN.* EBioMedicine, 2019. **43**: p. 473-486.

218. Zhang, M.Q., et al., *Neurobehavioural abnormalities induced by repeated exposure of neonatal rats to sevoflurane can be aggravated by social isolation and enrichment deprivation initiated after exposure to the anaesthetic.* Br J Anaesth, 2015. **115**(5): p. 752-60.

219. Zhang, X., et al., *In Vivo Monitoring of Sevoflurane-induced Adverse Effects in Neonatal Nonhuman Primates Using Small-animal Positron Emission Tomography.* Anesthesiology, 2016. **125**(1): p. 133-46.

220. Zhang, X., et al., *A lasting effect of postnatal sevoflurane anesthesia on the composition of NMDA receptor subunits in rat prefrontal cortex.* Int J Dev Neurosci, 2016. **54**: p. 62-69.

221. Zhang, Y., et al., *Cyclophilin D Contributes to Anesthesia Neurotoxicity in the Developing Brain.* Front Cell Dev Biol, 2019. **7**: p. 396.

222. Zhao, P., et al., *Isoflurane postconditioning improved long-term neurological outcome possibly via inhibiting the mitochondrial permeability transition pore in neonatal rats after brain hypoxia-ischemia.* Neuroscience, 2014. **280**: p. 193-203.

223. Zhao, P., et al., *Isoflurane preconditioning improves long-term neurologic outcome after hypoxic-ischemic brain injury in neonatal rats.* Anesthesiology, 2007. **107**(6): p. 963-970.

224. Zhao, S., et al., *The differential effects of isoflurane and sevoflurane on neonatal mice.* Sci Rep, 2020. **10**(1): p. 19345.

225. Zhao, X., et al., *Sevoflurane impairs learning and memory of the developing brain through post-transcriptional inhibition of CCNA2 via microRNA-19-3p.* Aging (Albany NY), 2018. **10**(12): p. 3794-3805.

226. Zhao, Y., K. Chen, and X. Shen, *Environmental Enrichment Attenuated Sevoflurane-Induced Neurotoxicity through the PPAR-gamma Signaling Pathway.* BioMed Research International, 2015(107149).

227. Zheng, S.Q., et al., *Sevoflurane causes neuronal apoptosis and adaptability changes of neonatal rats.* Acta Anaesthesiologica Scandinavica, 2013(9): p. 1167-1174.

228. Zheng, S.Q., et al., *[Effects of sevoflurane on brain neuroapoptosis and ability of long-term learning and memory in newborn rats].* Beijing Da Xue Xue Bao Yi Xue Ban, 2015. **47**(4): p. 674-8.

229. Zhong, T., et al., *Neonatal isoflurane exposure induces neurocognitive impairment and abnormal hippocampal histone acetylation in mice.* PLoS One, 2015. **10**(4): p. e0125815.

230. Zhou, B., et al., *Astroglial dysfunctions drive aberrant synaptogenesis and social behavioral deficits in mice with neonatal exposure to lengthy general anesthesia.* PLoS Biol, 2019. **17**(8): p. e3000086.

231. Zhou, H., et al., *Protective effect of FTY720 against sevoflurane-induced developmental neurotoxicity in rats.* Cell Biochem Biophys, 2013. **67**(2): p. 591-8.

232. Zhou, H., S. Li, and G. Wang, *Euxanthone Ameliorates Sevoflurane-Induced Neurotoxicity in Neonatal Mice.* Journal of molecular neuroscience : MN., 2019.

233. Zhou, X., et al., *Lithium Treatment Prevents Apoptosis in Neonatal Rat Hippocampus Resulting from Sevoflurane Exposure.* Neurochemical Research, 2016(8): p. 1993-2005.

234. Zhou, X., et al., *Dose-dependent effects of sevoflurane exposure during early lifetime on apoptosis in hippocampus and neurocognitive outcomes in Sprague-Dawley rats.* Int J Physiol Pathophysiol Pharmacol, 2016. **8**(3): p. 111-119.

235. Zhou, X., et al., *Aberrantly expressed long noncoding RNAs are involved in sevoflurane-induced developing hippocampal neuronal apoptosis: a microarray related study.* Metabolic Brain Disease, 2016(5): p. 1031-1040.

236. Zhou, X., et al., *Neonatal exposure to sevoflurane causes apoptosis and reduces nNOS protein expression in rat hippocampus.* Molecular Medicine Reports, 2012(3): p. 543-546.

237. Zhou, X., et al., *MicroRNA-34c is regulated by p53 and is involved in sevoflurane-induced apoptosis in the developing rat brain potentially via the mitochondrial pathway.* Molecular Medicine Reports, 2017(4): p. 2204-2212.

238. Zhou, Z.B., et al., *Subclinical concentrations of sevoflurane reduce oxidative stress but do not prevent hippocampal apoptosis.* Molecular Medicine Reports, 2016(1): p. 721-727.

239. Zhou, Z.B., et al., *Sevoflurane-induced down-regulation of hippocampal oxytocin and arginine vasopressin impairs juvenile social behavioral abilities.* J Mol Neurosci, 2015(1): p. 70-7.

240. Zhu, C., et al., *Isoflurane anesthesia induced persistent, progressive memory impairment, caused a loss of neural stem cells, and reduced neurogenesis in young, but not adult, rodents.* Journal of Cerebral Blood Flow and Metabolism, 2010. **30**(5): p. 1017-1030.

241. Zhu, J., et al., *Sevoflurane Induces Learning and Memory Impairment in Young Mice Through a Reduction in Neuronal Glucose Transporter 3.* Cell Mol Neurobiol, 2019.

242. Zhu, X., G.Q. Zhu, and L.C. Zhang, *Effects of different concentrations of sevoflurane on learning and memory ability in infantile rats and on the Tyr1472 phosphorylation of hippocampal NR2B. [Chinese].* Chinese Pharmacological Bulletin, 2013. **29**(12): p. 1717-1721.

243. Aldemir Şensoy, D., et al., *Effect of Isoflurane Exposure with Administration of Polyunsaturated Fatty Acids on Cognition in Developing Rats.* Turk J Anaesthesiol Reanim, 2020. **48**(6): p. 477-483.

244. Chen, B.Z., et al., *Repeated Sevoflurane Exposures in Neonatal Rats Increased the Brain Vulnerability to Future Stress Exposure and Resulted in Fear Extinction Deficit.* Neurotox Res, 2022. **40**(5): p. 1405-1414.

245. Chen, B.Z., et al., *Repeated Sevoflurane Exposure in Neonatal Rats Enhances the Sensitivity to Pain and Traumatic Stress Later in Juvenile Life.* Journal of Pain Research, 2022. **15**: p. 3171-3178.

246. Chen, M.K., et al., *Effect of Long-time Postnatal Exposure to Sevoflurane on Causing Attention-deficit/Hyperactivity Disorder in Rats. [Chinese].* Sichuan da xue xue bao, 2021: p. 207-215.

247. Chen, W., Z. He, and M. Jiang, *Anti-inflammatory, antioxidant and neuroprotection effect of thiopental sodium on isoflurane-induced cognitive dysfunction in rats.* International Journal of Pharmacology, 2021. **17**: p. 611-620.

248. Chen, Y., X. Gao, and H. Pei, *miRNA-384-3p alleviates sevoflurane-induced nerve injury by inhibiting Aak1 kinase in neonatal rats.* Brain Behav, 2022. **12**(7): p. e2556.

249. Chen, Y., Y. Xie, and H. Ni, *Effects of overexpression of Hsp70 in neural stem cells on neurotoxicity and cognitive dysfunction in neonatal mice under sevoflurane exposure.* Exp Brain Res, 2022. **240**(12): p. 3207-3216.

250. Chen, Y.R., et al., *Egr2 contributes to age-dependent vulnerability to sevoflurane-induced cognitive deficits in mice.* Acta Pharmacol Sin, 2022. **43**(11): p. 2828-2840.

251. Cui, L.Y., J. Zhang, and Y. Ying, *Protective effect of 3-Methyladenine on Neuronal Damage Induced by Sevoflurane in Young Mice. [Chinese].* Chinese Pharmaceutical Journal, 2021. **56**: p. 912-918.

252. Dai, J., et al., *Repeated neonatal sevoflurane induced neurocognitive impairment through NF-kappaB-mediated pyroptosis.* Journal of Neuroinflammation, 2021. **18**.

253. Demirgan, S., et al., *Intranasal levosimendan prevents cognitive dysfunction and apoptotic response induced by repeated isoflurane exposure in newborn rats.* Naunyn Schmiedebergs Arch Pharmacol, 2021. **394**(7): p. 1553-1567.

254. Fan, X.Y., G. Shi, and P. Zhao, *Neonatal Sevoflurane Exposure Impairs Learning and Memory by the Hypermethylation of Hippocampal Synaptic Genes.* Molecular Neurobiology, 2021. **58**: p. 895-904.

255. Fang, X., et al., *Melatonin attenuates spatial learning and memory dysfunction in developing rats by suppressing isoflurane-induced endoplasmic reticulum stress via the SIRT1/Mfn2/PERK signaling pathway.* Heliyon, 2022. **8**(9): p. e10326.

256. Gill, H. and A.E. Pickering, *The effects of xenon on sevoflurane anesthesia-induced acidosis and brain cell apoptosis in immature rats.* Paediatric Anaesthesia, 2021. **31**: p. 372-374.

257. Huang, C., et al., *PP2A-associated tau hyperphosphorylation was involved in sevoflurane induced neonatal neurotoxicity.* Psychopharmacology (Berl), 2022. **239**(9): p. 2799-2807.

258. Huang, H., et al., *Triiodothyronine attenuates neurocognitive dysfunction induced by sevoflurane in the developing brain of neonatal rats.* J Affect Disord, 2022. **297**: p. 455-462.

259. Huang, Y., et al., *Inhibiting PDE7A Enhances the Protective Effects of Neural Stem Cells on Neurodegeneration and Memory Deficits in Sevoflurane-Exposed Mice.* eNeuro, 2021. **8**(4).

260. Jian, W., et al., *Sex-specific effects of sevoflurane on cognitive function of postnatal rats. [Chinese].* Medical Journal of Chinese People's Liberation Army, 2021. **41**: p. 114-121.

261. Jiang, C., et al., *Expression Signature of lncRNAs and mRNAs in Sevoflurane-Induced Mouse Brain Injury: Implication of Involvement of Wide Molecular Networks and Pathways.* Int J Mol Sci, 2021. **22**(3).

262. Jiang, Y., et al., *Sevoflurane induces microRNA-18a to delay rat neurodevelopment via suppression of the RUNX1/Wnt/β-catenin axis.* Cell Death Discov, 2022. **8**(1): p. 404.

263. Jung, S., et al., *Tetraethylammonium chloride reduces anaesthetic-induced neurotoxicity in Caenorhabditis elegans and mice.* Br J Anaesth, 2022. **128**(1): p. 77-88.

264. Ke, W., X. Yu, and Y. Gao, *Neonatal exposure to sevoflurane caused learning and memory impairment via dysregulating SK2 channel endocytosis.* Sci Prog, 2021. **104**(3): p. 368504211043763.

265. Lee, J.R., et al., *Effect of dexmedetomidine on sevoflurane-induced neurodegeneration in neonatal rats.* Br J Anaesth, 2021. **126**(5): p. 1009-1021.

266. Lee, Y., et al., *General anesthesia activates the mitochondrial unfolded protein response and induces age-dependent, long-lasting changes in mitochondrial function in the developing brain.* NeuroToxicology, 2021. **82**: p. 1-8.

267. Li, C., et al., *Differential Effects of Sevoflurane Exposure on Long-Term Fear Memory in Neonatal and Adult Rats.* Mol Neurobiol, 2022. **59**(5): p. 2799-2807.

268. Li, H., et al., *MiR-191 downregulation protects against isoflurane-induced neurotoxicity through targeting BDNF.* Toxicology Mechanisms and Methods, 2021. **31**: p. 367-373.

269. Li, H., et al., *Sevoflurane-induced neuronal apoptosis in neonatal mice is prevented with intranasal administration of insulin.* Am J Transl Res, 2020. **12**(12): p. 8175-8184.

270. Li, L., et al., *Inhibitive Effect of Luteolin on Sevoflurane-Induced Neurotoxicity through Activation of the Autophagy Pathway by HMOX1.* ACS Chemical Neuroscience, 2021. **12**: p. 3314-3322.

271. Li, N., et al., *The role of depolarizing activation of Na(+)-Ca(2+) exchanger by oligodendrocyte progenitor cells in the effect of sevoflurane on myelination.* Life Sci, 2022. **308**: p. 120951.

272. Li, R., et al., *Sevoflurane Exposure in the Developing Brain Induces Hyperactivity, Anxiety-Free, and Enhancement of Memory Consolidation in Mice.* Front Aging Neurosci, 2022. **14**: p. 934230.

273. Liang, L., et al., *Sevoflurane-Induced Neurotoxicity in the Developing Hippocampus via HIPK2/AKT/mTOR Signaling.* Neurotox Res, 2022. **40**(3): p. 803-813.

274. Liang, L., et al., *Melatonin pretreatment alleviates the long-term synaptic toxicity and dysmyelination induced by neonatal Sevoflurane exposure via MT1 receptor-mediated Wnt signaling modulation.* Journal of Pineal Research, 2021. **71**.

275. Liao, Z., et al., *Regulation of CRMP2 by Cdk5 and GSK-3β participates in sevoflurane-induced dendritic development abnormalities and cognitive dysfunction in developing rats.* Toxicol Lett, 2021. **341**: p. 68-79.

276. Liao, Z., et al., *Inhibition of RhoA Activity Does Not Rescue Synaptic Development Abnormalities and Long-Term Cognitive Impairment After Sevoflurane Exposure.* Neurochemical Research, 2021. **46**: p. 468-481.

277. Lin, L., et al., *The role and mechanism of TLR4-siRNA in the impairment of learning and memory in young mice induced by isoflurane.* Adv Clin Exp Med, 2022. **31**(7): p. 769-780.

278. Liu, J., et al., *Sevoflurane induced neurotoxicity in neonatal mice links to a GSK3β/Drp1-dependent mitochondrial fission and apoptosis.* Free Radic Biol Med, 2022. **181**: p. 72-81.

279. Liu, M., et al., *Gut microbiota mediates cognitive impairment in young mice after multiple neonatal exposures to sevoflurane.* Aging (Albany NY), 2021. **13**(12): p. 16733-16748.

280. Liu, Y., et al., *TRPV1 Antagonist Prevents Neonatal Sevoflurane-Induced Synaptic Abnormality and Cognitive Impairment in Mice Through Regulating the Src/Cofilin Signaling Pathway.* Front Cell Dev Biol, 2021. **9**: p. 684516.

281. Luo, R.Y., et al., *Early-Life Multiple Sevoflurane Exposures Alleviate Long-term Anxiety-Like Behaviors in Mice via the proBDNF/ERK Pathway.* Molecular Neurobiology, 2021. **58**: p. 170-183.

282. Manzella, F.M., et al., *Neonatal Isoflurane Does Not Affect Sleep Architecture and Minimally Alters Neuronal Beta Oscillations in Adolescent Rats.* Front Behav Neurosci, 2021. **15**: p. 703859.

283. Mineshima, H., et al., *Comparative study on detectability of learning and memory disorder between two water maze tests commonly used in juvenile rat toxicity studies using isoflurane inhaled rat model.* Congenit Anom (Kyoto), 2022. **62**(3): p. 96-104.

284. Peng, L., et al., *Histone Deacetylase 2-Mediated Epigenetic Regulation is Involved in the Early Isoflurane Exposure-Related Increase in Susceptibility to Anxiety-Like Behaviour Evoked by Chronic Variable Stress in Mice.* Neurochem Res, 2021. **46**(9): p. 2333-2347.

285. Peng, L., et al., *Repeated Neonatal Isoflurane Exposure is Associated with Higher Susceptibility to Chronic Variable Stress-induced Behavioural and Neuro-inflammatory Alterations.* Neuroscience, 2021. **465**: p. 166-176.

286. Piao, M., et al., *Sevoflurane Exposure Induces Neuronal Cell Parthanatos Initiated by DNA Damage in the Developing Brain via an Increase of Intracellular Reactive Oxygen Species.* Front Cell Neurosci, 2020. **14**: p. 583782.

287. Qian, D., et al., *MiR-128-3p Attenuates the Neurotoxicity in Rats Induced by Isoflurane Anesthesia.* Neurotoxicity Research, 2022. **40**: p. 714-720.

288. Que, Y., et al., *Repeated isoflurane exposures of neonatal rats contribute to cognitive dysfunction in juvenile animals: the role of miR-497 in isoflurane-induced neurotoxicity.* Folia Histochem Cytobiol, 2021. **59**(2): p. 114-123.

289. Roque, P.S., et al., *Intranasal insulin rescues repeated anesthesia-induced deficits in synaptic plasticity and memory and prevents apoptosis in neonatal mice via mTORC1.* Sci Rep, 2021. **11**(1): p. 15490.

290. Sümer Coşkun, A., et al., *Does Resveratrol Prevent Sevoflurane Toxicity in Newborn Rats?* J Med Food, 2022. **25**(5): p. 557-563.

291. Sun, M., et al., *Dexmedetomidine and Clonidine Attenuate Sevoflurane-Induced Tau Phosphorylation and Cognitive Impairment in Young Mice via alpha-2 Adrenergic Receptor.* Anesthesia and Analgesia, 2021. **132**: p. 878-889.

292. Tang, X.L., et al., *Resveratrol ameliorates sevoflurane-induced cognitive impairment by activating the SIRT1/NF-κB pathway in neonatal mice.* J Nutr Biochem, 2021. **90**: p. 108579.

293. Tong, D., et al., *Sevoflurane-Induced Neuroapoptosis in Rat Dentate Gyrus Is Activated by Autophagy Through NF-κB Signaling on the Late-Stage Progenitor Granule Cells.* Front Cell Neurosci, 2020. **14**: p. 590577.

294. Useinovic, N., et al., *Systemic inflammation exacerbates developmental neurotoxicity induced by sevoflurane in neonatal rats.* Br J Anaesth, 2022. **129**(4): p. 555-566.

295. Wali, B., et al., *Prophylactic progesterone prevents adverse behavioural and neurocognitive effects of neonatal anaesthesia exposure in rat.* Br J Anaesth, 2022. **128**(2): p. 301-310.

296. Wang, D.W., et al., *Protective effect of betulinic acid against isoflurane induced neuronal damage in developing rats. [Chinese].* Chinese Journal of Pharmacology and Toxicology, 2021. **35**: p. 438-443.

297. Wang, F., et al., *Sevoflurane induces inflammation of microglia in hippocampus of neonatal rats by inhibiting Wnt/β-Catenin/CaMKIV pathway.* J Pharmacol Sci, 2021. **146**(2): p. 105-115.

298. Wang, Q., et al., *Sevoflurane-Induced Apoptosis in the Mouse Cerebral Cortex Follows Similar Characteristics of Physiological Apoptosis.* Front Mol Neurosci, 2022. **15**: p. 873658.

299. Wang, X., et al., *Effects of Sevoflurane and Isoflurane Anaesthesia on Long-Term Cognitive Function and Hippocampal Inflammatory Response in Neonatal Mice.* Acta Medica Mediterranea, 2022. **38**: p. 3239-3243.

300. Wang, Y., et al., *Neonatal exposure to sevoflurane induces adolescent neurobehavioral dysfunction by interfering with hippocampal glycerophoslipid metabolism in rats.* Cerebral cortex, 1991.

301. Wei, K., et al., *Bumetanide attenuates sevoflurane-induced neuroapoptosis in the developing dentate gyrus and impaired behavior in the contextual fear discrimination learning test.* Brain Behav, 2022. **12**(11): p. e2768.

302. Wen-Yuan, W., et al., *ZBP1 senses mitochondrial DNA to induce RIPK3/MLKL-driven necroptosis in developmental sevoflurane neurotoxicity.* Neuroscience., 2022. **9**.

303. Wu, Y., et al., *Maresin 1 alleviates sevoflurane-induced neuroinflammation in neonatal rats via JAK2/STAT3/IL-6 pathways.* Int Immunopharmacol, 2022. **108**: p. 108912.

304. Wu, Y., et al., *Differential epitranscriptome and proteome modulation in the brain of neonatal mice exposed to isoflurane or sevoflurane.* Cell Biol Toxicol, 2022.

305. Xu, L., et al., *lncRNA Xist regulates sevoflurane-induced social and emotional impairment by modulating miR-98-5p/EDEM1 signaling axis in neonatal mice.* Molecular Therapy - Nucleic Acids, 2021. **24**: p. 939-950.

306. Xu, R., et al., *RIPK1/RIPK3-Mediated Necroptosis is Involved in Sevoflurane-Induced Neonatal Neurotoxicity in the Rat Hippocampus.* Cell Mol Neurobiol, 2022. **42**(7): p. 2235-2244.

307. Yang, Y., et al., *Testosterone attenuates sevoflurane-induced tau phosphorylation and cognitive impairment in neonatal male mice.* Br J Anaesth, 2021. **127**(6): p. 929-941.

308. Yang, Y., et al., *Dexmedetomidine-mediated Wnt pathway inhibits sevoflurane-induced cognitive impairment in neonatal rats. [Chinese].* Acta Academiae Medicinae Sinicae, 2021. **43**: p. 235-246.

309. Yang, Z., et al., *Dexmedetomidine Diminishes, but Does Not Prevent, Developmental Effects of Sevoflurane in Neonatal Rats.* Anesth Analg, 2022. **135**(4): p. 877-887.

310. Yılmaz, H., et al., *Acutely increased aquaporin-4 exhibits more potent protective effects in the cortex against single and repeated isoflurane-induced neurotoxicity in the developing rat brain.* Toxicol Mech Methods, 2022: p. 1-14.

311. Yu, Y., et al., *Effects of toxic apolipoprotein E fragments on Tau phosphorylation and cognitive impairment in neonatal mice under sevoflurane anesthesia.* Brain Behav, 2022. **12**(8): p. e2702.

312. Zeng, S., et al., *Role of GABA(A) receptor depolarization-mediated VGCC activation in sevoflurane-induced cognitive impairment in neonatal mice.* Front Cell Neurosci, 2022. **16**: p. 964227.

313. Zhang, P., et al., *Mitochondria-Related Ferroptosis Drives Cognitive Deficits in Neonatal Mice Following Sevoflurane Administration.* Front Med (Lausanne), 2022. **9**: p. 887062.

314. Zhang, Q., et al., *IL-17A deletion reduces sevoflurane-induced neurocognitive impairment in neonatal mice by inhibiting NF-κB signaling pathway.* Bioengineered, 2022. **13**(6): p. 14562-14577.

315. Zhang, W., et al., *Prolonged sevoflurane exposure causes abnormal synapse development and dysregulates beta-neurexin and neuroligins in the hippocampus in neonatal rats.* J Affect Disord, 2022. **312**: p. 22-29.

316. Zhang, Y., et al., *Dexmedetomidine attenuates sevoflurane-induced neurocognitive impairment through alpha2-adrenoceptors.* Molecular Medicine Reports, 2021. **23**: p. 1-8.

317. Zhang, Y., et al., *Overexpression of miR-133b protects against isoflurane-induced learning and memory impairment.* Exp Ther Med, 2021. **22**(5): p. 1207.

318. Zhao, J., et al., *Repeated exposure to sevoflurane in neonatal rats impairs cognition in adulthood via the PKA-CREB-BDNF signaling pathway.* Exp Ther Med, 2021. **22**(6): p. 1442.

319. Zhao, W., et al., *Disruption of hippocampal P2RX2/CaMKII/NF-κB signaling contributes to learning and memory impairment in C57BL/6 mice induced by surgery plus anesthesia in neonatal period.* Biomed Pharmacother, 2022. **149**: p. 112897.

320. Zhao, X., et al., *Repeated Neonatal Exposure to Sevoflurane Induces Age-Dependent Impairments in Cognition and Synaptic Plasticity in Mice.* Dev Neurosci, 2022. **44**(3): p. 153-161.

321. Zhao, X.P., et al., *Early-life sevoflurane exposure impairs fear memory by suppressing extracellular signal-regulated kinase signaling in the bed nucleus of stria terminalis GABAergic neurons.* Neuropharmacology, 2021. **191**: p. 108584.

322. Zhong, Y., et al., *Multiple exposures to sevoflurane across postnatal development may cause cognitive deficits in older age.* Pediatr Res, 2022.

323. Zhu, Y., et al., *Knockdown of UAF1 alleviates sevoflurane-induced cognitive impairment and neurotoxicity in rats by inhibiting pro-inflammatory signaling and oxidative stress.* J Toxicol Sci, 2022. **47**(9): p. 349-357.

324. Zuo, C., et al., *Isoflurane and Sevoflurane Induce Cognitive Impairment in Neonatal Rats by Inhibiting Neural Stem Cell Development Through Microglial Activation, Neuroinflammation, and Suppression of VEGFR2 Signaling Pathway.* Neurotox Res, 2022. **40**(3): p. 775-790.
