# Supplementary figures and images for: Exposure to halogenated ethers causes neurodegeneration and behavioural changes in young healthy experimental animals: a systematic review and meta analyses
Source: Sci Rep. 2023 May 18;13:8063. doi: 10.1038/s41598-023-35052-4 (PMC10195874; doi:10.1038/s41598-023-35052-4)

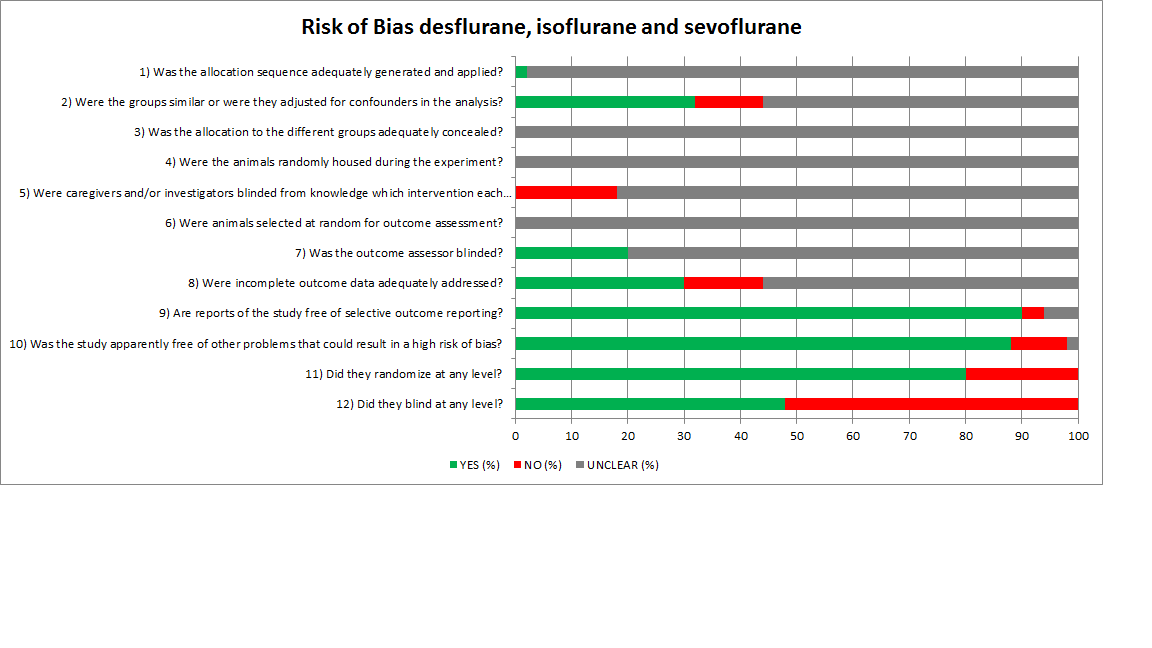

Supplement: Supplementary file 8 — Supplementary Information 8. [file 41598_2023_35052_MOESM8_ESM.tif]
