# Supplementary material for: Exposure to halogenated ethers causes neurodegeneration and behavioural changes in young healthy experimental animals: a systematic review and meta analyses
Source: Sci Rep. 2023 May 18;13:8063. doi: 10.1038/s41598-023-35052-4 (PMC10195874; doi:10.1038/s41598-023-35052-4)
Supplement: Supplementary file 10 — Supplementary Information 10. [file 41598_2023_35052_MOESM10_ESM.docx]

**Supplemental file 9:** results of Duval and Tweedie’s trim and fill analysis and the Egger’s regression test

|  |  | Egger’s regression | Trim and fill |
| --- | --- | --- | --- |
| Sevoflurane | Caspase-3 | p = 0.47 | Imputed studies: 0 |
|  | TUNEL | p = 0.49 | Imputed studies: 0 |
|  | MWM | p=0.23 | Imputed studies: 0 |
|  | Contextual Fear Conditioning Test | Too few studies |  |
|  | Cued Fear Conditioning Test | p = 0.32 | Imputed studies: 0 |
|  | Elevated Plus Maze | Too few studies |  |
|  | Open Field Test | p = 0.54 | Imputed studies: 0 |
| Isoflurane | Caspase-3 | p = 0.174 | Imputed studies: 0 |
|  | TUNEL | p = 0.45 | Imputed studies: 0 |
|  | MWM | p= 0.08 | Imputed studies: 0 |
|  | Contextual Fear Conditioning Test | Too few studies |  |
|  | Cued Fear Conditioning Test | Too few studies |  |
|  | Elevated Plus Maze | Too few studies |  |
|  | Open Field Test | Too few studies |  |
